# Advanced deep-learning model for temporal-dependent prediction of dynamic behavior of AC losses in superconducting propulsion motors for hydrogen-powered cryo-electric aircraft

**DOI:** 10.1038/s44172-025-00554-8

**Published:** 2025-12-17

**Authors:** Shahin Alipour Bonab, Frederick Berg, Wenjuan Song, Alexandre Colle, Mohammad Yazdani-Asrami

**Affiliations:** 1 CryoElectric Research Lab, Propulsion, Electrification & Superconductivity Group, Autonomous Systems and Connectivity Division, James Watt School of Engineering, University of Glasgow, G12 8QQ, Glasgow, UK; 2 Airbus X-Labs, Taufkirchen, Germany; 3 Airbus UpNext, Toulouse, France

**Keywords:** Electrical and electronic engineering, Aerospace engineering

## Abstract

Superconducting motors offer high power density, compactness, and efficiency for hydrogen-powered cryo-electric aircraft, but AC operation in cryogenic temperatures produces thermal losses that must be estimated accurately and rapidly at the design stage to optimize efficiency, minimize cryogenic heat load, and maximize specific power density. Traditional modeling approaches fall short—Finite-element is too slow/costly for system-level models, analytical models and look-up tables lack accuracy/flexibility, and earlier intelligent models gave only cycle-averaged (static) losses. Here we demonstrate AI can rapidly and accurately predict dynamic AC losses for superconducting propulsion motors. Using a large dataset of motor configurations, our AI-driven approach both predicts cycle-averaged and time-dependent morphology of instantaneous AC loss waveforms across various operating conditions and generalizes to unseen designs. Integrated into system-level model-based design, these AI-surrogate models enable rapid model trials, compliance checks, and the discovery of integration issues within simulated environments before propulsion motor deployment. Our deep learning-based model achieves a prediction time of less than 9 ms with a 99.97% accuracy (R^2^), making it suitable for system-level modeling of electric powertrains in hydrogen-powered cryo-electric aircraft. Furthermore, we benchmarked 14 AI and 2 mathematical fitting techniques for estimating average AC losses, providing comparative performance analysis. The results highlight that AI-based surrogate models enable high-accuracy, low-latency loss predictions to achieve optimal performance in superconducting propulsion motors in aircraft powertrain design.

## Abstract (web summary)

Shahin Alipour Bonab and colleagues proposed and developed AI surrogate models that can predict time-dependent AC losses of superconducting motor of future hydrogen-powered cryo-electric aircraft. Benefiting of ultra-fast predictions, the model will be used in system-level model for aircraft propulsion system in the context Airbus CryoProp project.

## Introduction

The aviation industry is proactively seeking ways to reduce emissions by transitioning to cleaner and more sustainable air travel^1^ and is exploring means to achieve net-zero emission commercial aviation, for example, in the Airbus ZEROe project that aims to use hydrogen fuel cells to generate propulsive power^2^. The concept of cryo-electric aircraft, which integrates liquid hydrogen (LH_2_) as fuel and the coolant for superconducting components, is emerging as a promising solution for achieving Net-Zero targets in the aviation sector^3–6^. Industrial demonstrator projects like Airbus UpNext’s ASCEND^7^ and Cryoprop and NASA’s CHEETA^8^ are pioneering these efforts, picturing future aircraft propulsion systems that utilize the benefits of superconducting technology^9,10^ to dramatically improve efficiency, increase specific power density, and reduce aviation’s carbon footprint^11–13^.

Superconductors are recognized for their ability to conduct extremely large currents with zero resistance in the DC mode^14^; however, under AC conditions, they produce AC losses^15–20^. Although these losses are minor in small-scale setups, they become substantial at the megawatt power levels needed for aircraft propulsion^21^. Also, it is worth noting that these losses are released at cryogenic temperature within the motor cryostat^22,23^ and must be dissipated to ambient temperature out of the cryostat (to maintain motor operating temperature within the designed range), thus, complex cooling systems would be required, which increases the overall weight and size of the system, impacting the specific power density of the propulsion motor and reducing the overall efficiency of aircraft powertrain^21–25^. On the other hand, the cooling capacity to absorb and remove heat is limited either due to the weight and cooling capacity of the cryocooler or due to the limited amount of LH_2_ that is carried onboard aircraft during a flight. Therefore, the AC loss of the superconducting motors must be traded off with motor size and weight by optimization of their design and testing of their functionality in conjunction with other powertrain components^26,27^ in system-level studies^28,29^.

**Fig. 1:**
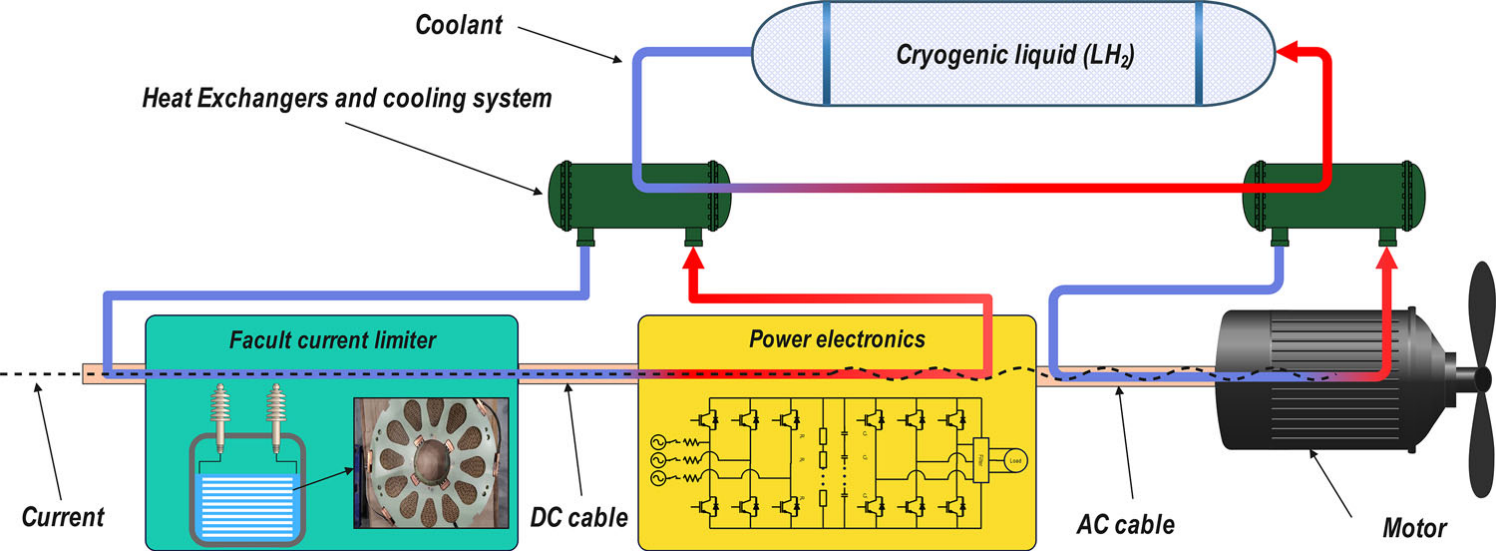
An illustration of a cryo-electric propulsion system.

A system-level modeling approach, illustrated in Fig. 1, is essential for capturing how the AC losses of the superconducting propulsion motor, cooling capacity, component mass, and propulsion demand collectively shape the final propulsion system design, given their close interdependencies^30–32^. Within this holistic approach, an integrated time-domain simulation framework is implemented to link each powertrain sub-model (motors, cryogenics, converters, fault current limiters, power cables, etc.), allowing system-level design trade-offs—such as higher rotational speed or increased current density—to be assessed^33,34^. Through this method, the purpose is to minimize AC losses of superconducting motors in combination with other powertrain objectives, leading to an optimized balance of weight, power density, and efficiency across the propulsion systems^35^.

Currently, superconducting component models in a system-level study are mostly developed based on analytical models and look-up tables in MATLAB Simulink that inherently suffer from sparse and limited experimental datasets, have low speed of computation, and lack adaptability to dynamic and transient scenarios^36^. Due to the complexity of experimental tests necessary for testing superconducting components at cryogenic temperatures (e.g., in LH_2_), providing enough experimental data for model validation can be expensive and time-consuming. In addition, currently used numerical models—mostly based on finite element analysis (FEA)—while offering high calculation accuracy, are not compatible with system-level models that require fast predictions of individual component models^37^. System-level models informed by FEA results generally require reparameterization whenever there is a design change, which can require time-consuming^38–41^ reruns of the FEA models.

To address these issues, surrogate models based on artificial intelligence (AI) techniques have gained substantial attention in recent years within the superconducting research community^42–48^. AI surrogate models can effectively capture the underlying patterns of data sourced from sparse experimental results or limited computationally expensive numerical simulations. These models can intelligently generalize the available data to cover a broader range of operating conditions, including scenarios outside the trained range, which the propulsion system of electric aircraft may encounter during flight. Furthermore, AI models can predict multiple outputs simultaneously^49^, reducing latency when utilized in system-level models and making them a viable tool for designing more efficient superconducting systems. Looking into the literature, only a handful of efforts have been made to predict AC losses of superconductors using AI models. In ref. ^50^, a hybrid model combining analytical scaling laws and Artificial Neural Networks (ANNs) has been presented to predict AC losses in superconducting round filaments under elliptical magnetic fields and transport currents. The model is trained using data generated through FEA simulations based on the H-formulation^51,52^ and achieves high accuracy with substantially reduced computation time compared to traditional methods. The results show that the hybrid model can predict AC losses with an average error of about 3.12%. In ref. ^53^, it was showcased that an AI surrogate model can predict AC losses in superconducting tapes under non-sinusoidal current waveforms with very high accuracy. Benchmarking different AI techniques on the same data, the radial basis function neural network model demonstrated the highest accuracy with an *R*-value near 1. Similarly, in ref. ^54^, an ANN-based model for the prediction of AC loss in REBCO lap joints was developed. As in refs. ^50,53^, the authors used a numerical model to generate training data for their ANN model to enhance prediction efficiency and drastically reduce computation time compared with the FEA model.

**Fig. 2:**
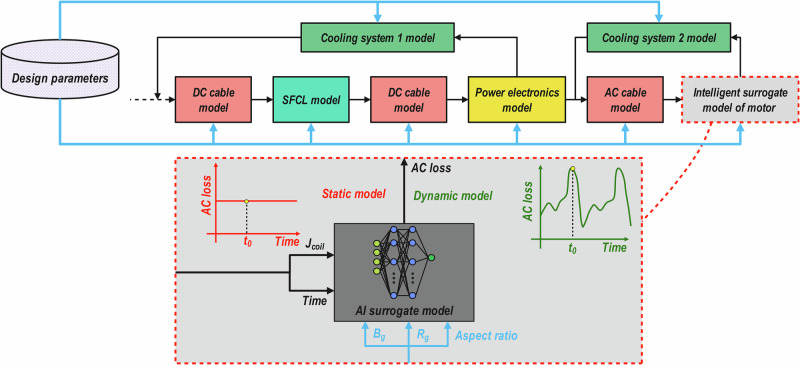
Data flow chart within a simple system-level model and the role of the artificial intelligence (AI) surrogate model to predict motor performance. While the model in both static and dynamic models receives a time signal, the predicted values of the static model do not change over time; however, dynamic models provide an accurate value of alternative current (AC) loss at any instance in time.

While these models demonstrate the potential use of AI techniques for developing AC loss predictor models in superconducting filaments, tapes, and joints, there is a lack of an AI surrogate model at the component and system levels focusing on superconducting propulsion motors. Also, these studies only focus on the average value of AC losses over a period (static AC loss prediction), making their model unable to predict time-dependent AC losses. The differences between these two model types have been shown in Fig. 2. This paper aims to develop an AI surrogate model for a superconducting motor that can accurately estimate the morphology of AC loss waveforms. This is crucial for applying the surrogate model in system-level models, where time-dependent simulations are required to obtain reliable results that account for the system’s dynamic behavior, rather than relying on static average values. Static AC loss modeling is crucial for simulations of longer periods, whereas the AC loss waveform being available in system-level models through a parametric surrogate model means that trade studies on more complex aspects of motor control, filtering, fault protection, and PWM for superconducting machines can be made in a way that was previously unachievable. For this purpose, a slot model of a superconducting motor has been simulated based on *T*–*A* formulation FEA in COMSOL Multiphysics. This FEA model has been used to generate the dataset for our proposed AI surrogate models for AC loss prediction. To ensure a proper AI algorithm is chosen for the prediction of AC losses, a comprehensive benchmarking of different AI techniques was provided, where 14 machine learning techniques were benchmarked and compared with simple mathematical fitting techniques. This approach also facilitates the simulation of difficult or expensive scenarios, hardware-in-the-loop validation, and early exploration of novel motor design/configuration cases. Additionally, AI-driven reduced-order modeling accelerates slow, high-fidelity simulations, allowing designers to iterate quickly while having high accuracy. At the end, we analyzed the best model’s performance on the prediction of AC losses for the motor configurations outside the trained range of design parameters.

The workflow of this paper’s research is illustrated in Fig. S1 in the supplementary information. First, a brief overview of the FEA slot model used and the sensitivity analysis on design parameters of the motor is explained in the section “Methods”. Next, the architecture of the AI techniques, along with the performance index metrics, is explained. In the section “Results and discussion”, first, a comprehensive benchmark of the various AI models that predict static AC loss (temporally independent) is presented and compared with non-AI fitting models. Then, the dynamic AC loss surrogate models and their performance under testing data are presented. After that, both models were tested under different scenarios to examine their generalizability across new data. Finally, the developed model was implemented into the Simulink environment to predict AC losses.

## Methods

### *T*–*A* formulation FEA for motor modeling: data generation

A slot model of a partially superconducting motor (with superconducting armature windings) was developed in the COMSOL environment using the *T*–*A* formulation to accurately evaluate the motor’s AC losses under various design and operating conditions (e.g., geometries, electrical loading, magnetic fields/loading, etc.). The simulated motor is a direct-drive one that will not be connected to any gearboxes. Therefore, the number of poles is high, and the slot pitches are small, which enables the modeling of the model without considering a moving mesh part, but still gains relatively accurate results. The slot model concept allows a consequent reduction in computation time while maintaining a good level of accuracy. It is defined by

- *Linearization of the problem in the Cartesian coordinate*: The assumption is representative when the slot pitch is small compared to the motor perimeter (this is the case for every motor sized for this application).

- *No rotating mesh*: The movement of the magnets is replaced by a linear source of magnetic vector potential in the airgap.

The source has the form as Eq. (1):

1 $${A}_{z}={A}_{0}\sin \left(\frac{\pi x}{{T}_{{{{\rm{p}}}}}}-\omega t\right)$$

and ${A}_{0}$ can be evaluated from Eq. (2):

2 $${A}_{0}=\frac{{B}_{{{{\rm{g}}}}}}{\pi .{T}_{{{{\rm{p}}}}}}$$

In Eq. (2), ${B}_{{{{\rm{g}}}}}$ is the maximal magnetic flux density generated by the magnets in the air-gap in $T$, ${T}_{{{{\rm{p}}}}}$ is the motor pole pitch in $m$ and $\omega$ is the electrical pulsation in rad/s.

The *T*–*A* formulation is a numerical method that is commonly used in the high temperature superconductors (HTS) modeling community, which employs two different potentials: the current vector potential *T* within superconducting regions and the magnetic vector potential *A* in non-conducting regions like air^55^. In superconducting areas, *T* is used to calculate the current density (*J*), while *A* is used to determine the magnetic flux density (*B*) in the surrounding space^56^. This dual-potential approach allows for efficient computation by focusing computational resources on the superconducting regions where nonlinearities are notable.

Mathematically, *T* and *A* are defined as

3 $$J=\nabla \times T$$

4 $$B=\nabla \times A$$

where *A* can be evaluated from the *A* formulation as an Eq. (5):

5 $${\nabla }^{2}A=-{\mu }_{r}{\mu }_{0}J$$

In this equation, ${\mu }_{{{{\rm{r}}}}}$ is the relative permeability and ${\mu }_{0}$ is the vacuum permeability.

**Fig. 3:**
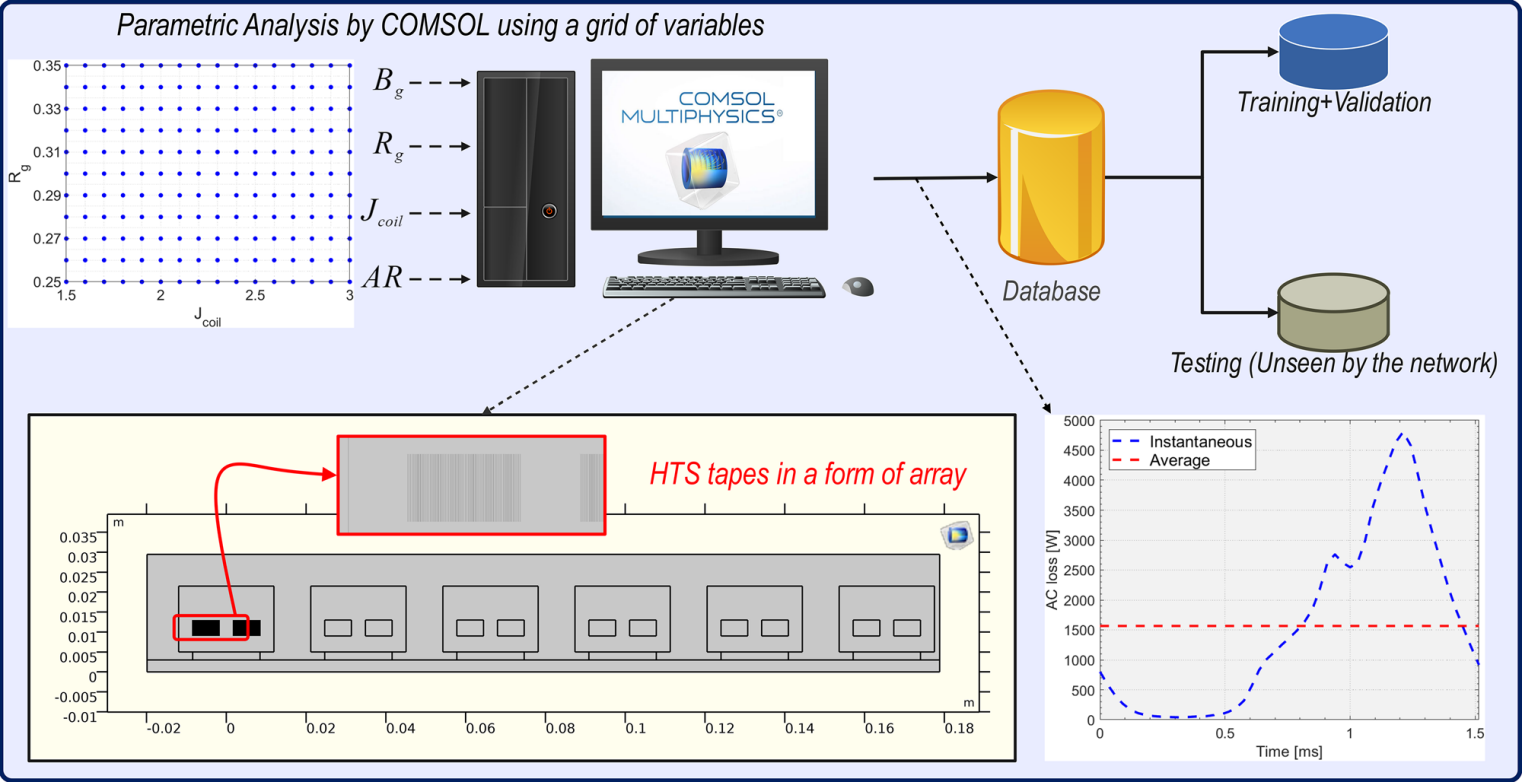
Schematic of the process of data generation and pre-processing for AI modeling. The yellow box demonstrates the geometry of the simulated slot model of the superconducting motor. In the bottom right, the figure shows how the AC loss value differs between the instantaneous data to the average data.

The *T* is also evaluated using the *T* formulation as shown in Eq. (6):

6 $$\nabla \times \rho \nabla \times T=-\frac{\partial B}{\partial t}$$

where *B* is evaluated from Eq. (4). In the 2D *T*–*A* formulation model, superconducting tapes are represented by a line, and the current density is restricted to flow in only one direction. The assumption is accurate due to the high aspect ratio of the HTS-coated tapes. SC coil is represented by an array of those lines with a number that depends on the number of tapes in parallel per turn and the number of turns (shown in Fig. 3).

To model the physical behavior of type II superconductors, the power law model is used. It gives the relation between the electric field, $E$ and the current density, $J$ with

7 $$E\left(J\right)=\rho \left(J\right)J$$

8 $$\rho (J)=\frac{{E}_{{{{\rm{c}}}}}}{{J}_{{{{\rm{c}}}}}(B)}{\left(|\frac{J}{{J}_{{{{\rm{c}}}}}(B)}|\right)}^{n-1}$$

where $E$ is the electric field intensity, $\rho \left(J\right)$ is the electrical resistivity, and *n* is the index value of the superconducting material. Also, the inhomogeneity of the critical current is applied to the model, and thus *J* is dependent on *B*. This dependence is implemented by interpolating the data provided by the Robinson Research Institute^57–59^. By solving the above equations with the time-series solver in COMSOL, the distribution of *J* and *E* within the superconducting parts of the motor over one working cycle is evaluated. To provide an example, Fig. S2 is provided in the supplementary information, which shows how the magnetic field within the motor geometry and electrical field, and normalized *J*(*J*/*J*_c_) within the superconducting parts are distributed. These contours were directly captured from the COMSOL model at the instance of three-quarters of the working cycle for the motor configuration of AR = 0.379, *R*_g_ = 0.25 m, *B*_g_ = 0.7 T, and *J*_coil_ = 1.5e8 A/m^2^ where these variables represent Aspect Ratio, air-gap radius, magnetic flux density, and current density of coil, respectively. Aspect ratio is defined as the ratio between the air-gap diameter and the active length of the motor. Using Eq. (9), the instantaneous AC loss for each time step can be evaluated.

9 $$Q={\int }_{{t}_{0}}^{{t}_{0}+\delta t}{{{\rm{d}}}}t{\int }_{S}E.J{{{\rm{d}}}}s$$

Now, using the developed model, the instantaneous AC loss of the motor can be evaluated for a given motor geometry and working condition. By performing a parametric sweep on the *B*_g_, *R*_g_, and *J*_coil_, giving the motor diameter over its length within the defined range of parameters, the required dataset for the intelligent surrogate model is constructed. The range of each parameter and the resulting AC loss (both static and dynamic) have been summarized in Table S1 provided in supplementary information. These four specific parameters and their representative ranges are defined in the collaborative research project between Airbus and the University of Glasgow in the context of CryoProp project, determined based on the physical constraints and design specifications of the motor to ensure the data’s relevance to the system’s performance. These parameters are, in fact, the inputs that will later be fed into the model for the prediction of the AC losses in the system-level model, which will be discussed at the end of the “Results” section.

A total number of 2816 propulsion motor case studies with different combinations of these parameters were simulated, and their AC loss waveform over one cycle was exported. Then, for the static model, the average value of each combination was evaluated, which resulted in a matrix size of 2816*5 for the generated data. For the dynamic model, by using all AC values for 51 time-steps within one working half-cycle, a massive matrix of size 143,616*6 (time was added into the matrix as an independent variable) was constructed for use in the next steps. Once the database is constructed, the data will be split into two sets: training and validation (80% of the total data), and testing (20% of the total data). This approach ensures that the testing portion of the data is not used during AI model development, thus providing a valid measure of the AI model’s performance. Figure 3 illustrates the above steps to facilitate understanding of the steps taken before starting the AI modeling.

### Overview of used AI techniques architectures

Each of the AI regression techniques employs different architectures to model relationships between input variables and target outputs. As the selection of a suitable method for each case study is paramount to ensure an accurate and fast surrogate model is developed, an extensive benchmarking approach has been taken in this paper. In the following, the implemented AI modeling techniques have been introduced, and an overview of their concept has been presented; however, as it is not an exhaustive overview presentation, for more information regarding the algorithms and their implementation the readers are referred to the references within each section and^60–62^. Also, Fig. S3 in the supplementary information provides a visual presentation of how each technique works. A general detailed algorithm for the development of the surrogate models has been provided as Algorithm S1 in the supplementary information.

### Feedforward neural network (FFNN)

FFNNs are the fundamental form of ANNs, consisting of an input layer, one or more hidden layers of neurons, and an output layer. Information moves in one direction from inputs to outputs; there are no feedback connections^63–65^. Each neuron performs a weighted sum of its inputs and passes it through an activation function^66^. An FFNN with one hidden layer models a function $f:x\mapsto \hat{y}$ can mathematically be written as^63^

10 $${y}_{{{{\rm{p}}}}}={f}^{0}\left(\displaystyle {\sum }_{j=1}^{n}{\omega }_{i}^{0}{x}^{i}{f}_{j}^{{{{\rm{H}}}}}\left(\displaystyle {\sum }_{i=1}^{n}{\omega }_{{ji}}^{{{{\rm{H}}}}}{x}_{i}\right)\right)$$

where ${f}^{0}$ and ${f}_{j}^{{{{\rm{H}}}}}$ designate the output layer activation function and the hidden layer activation function, respectively. Considering the addition of bias to both the input layer and the hidden layer, Eq. (11) turns into:

11 $${y}_{{{{\rm{p}}}}}={f}^{0}\left({\omega }^{{{{\rm{b}}}}}+\displaystyle {\sum }_{j=1}^{n}{\omega }_{i}^{0}{x}^{i}{f}_{j}^{{{{\rm{H}}}}}\left({\omega }_{i}^{0}+\displaystyle {\sum }_{i=1}^{n}{\omega }_{{ji}}^{{{{\rm{H}}}}}{x}_{i}\right)\right)$$

where ${\omega }_{j}^{{{{\rm{H}}}}}$ and ${\omega }^{{{{\rm{b}}}}}$ indicate the respective weights from bias to the hidden layer and output layer^63^. More details about FFNN can be found in the cited ref. ^67^.

### Cascade-forward neural network (CFNN)

**Fig. 4:**
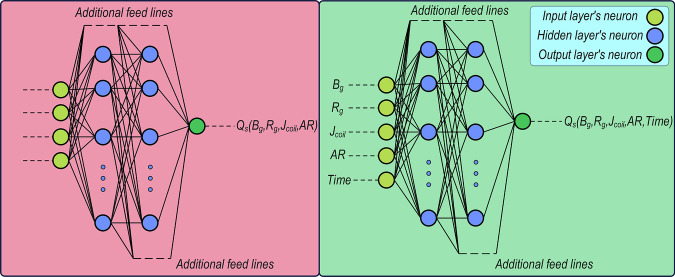
Schematic of the architecture of CFNN models. Prediction of static AC loss using *B*_g_, *R*_g_, *J*_coil_, and AR (left) and dynamic AC losses using *B*_g_, *R*_g_, *J*_coil_, AR, and time.

CFNNs are an extension of FNNs that include additional connections from the input and previous layers to all subsequent layers. In a CFNN, each hidden layer neuron receives not only the outputs of the previous layer but also the original inputs (and potentially outputs of all earlier layers)^68–70^. This unique connection has been illustrated in Fig. 4. This provides direct input influence on deeper layers, potentially improving learning of complex relationships. Also, architecture helps the network to learn faster in most cases (in comparison to FFNN), as it provides more direct paths for information/data to flow through the network. Furthermore, it has been shown that it has great potential to be used for time-series prediction tasks, where the aim of the model is to learn the complex signal form of the data^71^.

Mathematically, the output of the network can be expressed as Eq. (12)^72^:

12 $${y}_{{{{\rm{p}}}}}=\displaystyle {\sum }_{i=1}^{n}{f}^{i}{\omega }_{i}^{0}{x}^{i}+{f}^{0}\left(\displaystyle {\sum }_{j=1}^{n}{\omega }_{i}^{0}{x}^{i}{f}_{j}^{{{{\rm{H}}}}}\left(\displaystyle {\sum }_{i=1}^{n}{\omega }_{{jh}}^{{{{\rm{H}}}}}{x}_{i}\right)\right)$$

where ${f}^{i}$ and ${f}_{j}^{{{{\rm{H}}}}}$ designate the output layer activation function and the hidden layer activation function, respectively. By adding bias to both the input layer and the hidden layers, Eq. (12) will change to^72^:

13 $${y}_{{{{\rm{p}}}}}=\displaystyle {\sum }_{i=1}^{n}{f}^{i}{\omega }_{i}^{0}{x}^{i}+{f}^{0}\left({\omega }^{{{{\rm{b}}}}}+\displaystyle {\sum }_{j=1}^{n}{\omega }_{i}^{0}{x}^{i}{f}_{j}^{{{{\rm{H}}}}}\left({\omega }_{i}^{0}+\displaystyle {\sum }_{i=1}^{n}{\omega }_{{jh}}^{{{{\rm{H}}}}}{x}_{i}\right)\right)$$

Once the network has been constructed, an optimizer is used to find the optimal values of the weight and bias factors within the network that result in the minimal error compared to the actual values of AC losses. The optimizer that is used in this work is Levenberg–Marquardt, which is a strong algorithm that uses the second-order of the error and tries to minimize it as the objective function. More information on this algorithm can be found in refs. ^73–75^. A detailed algorithm for the development of the model has been provided as Algorithm S2 in the supplementary information.

### Generalized regression neural network (GRNN)

GRNN is a type of ANN primarily used for regression tasks. It is based on the principles of non-parametric regression, specifically the kernel density estimation technique^76^. The architecture of a GRNN consists of four layers: input, pattern, summation, and output layers. The input layer feeds the data into the network, and the pattern layer computes the distances between the input data and training examples using a radial basis function (typically a Gaussian function). The summation layer (numerator) collects these results with a weighted summation of outputs of the previous layer, the denominator computes the sum of the Gaussian kernels, and the output layer produces the final prediction by taking a weighted sum of the responses from the pattern layer^77,78^. Mathematically, the numerator and denominator nodes’ output can be written as^77–79^

14 $$S={\sum }_{j=1}^{J}{y}_{j}K\left({x,x}_{j}\right)$$

15 $$D={\sum }_{j=1}^{J}K\left({x,x}_{j}\right)$$

where *K* is the kernel function and is evaluated from Eq. (16):

16 $$K\left({x,x}_{j}\right)=exp \left(\frac{{||x}-{x}_{j}{||}}{-2{\alpha }^{2}}\right)$$

In this equation, $\alpha$ is a smoothing parameter whose value needs to be tuned by optimization techniques. The final prediction of the network is finally calculated using the equation below:

17 $$Y(x)=\frac{S}{D}$$

GRNNs excel at approximating continuous functions and are particularly effective when dealing with noisy or complex datasets. Unlike FFNNs, which rely on backpropagation and gradient descent to learn weights, GRNNs do not involve iterative training. Instead, GRNNs make predictions based on the entire training dataset without requiring weight updates or learning. The GRNN model works by using the training examples directly to estimate the conditional probability of the output given the input, and it produces an output based on how similar a new input is to the examples in the training set. The network’s performance is largely influenced by the spread or smoothing parameter of the radial basis function, which determines the width of the neighborhood used to make predictions^80^.

### Boosting methods

Gradient boosting is a family of AI techniques that iteratively combines multiple weak learners, usually decision trees, to minimize a chosen loss function. Each subsequent learner attempts to correct the errors made by the ensemble so far, resulting in a powerful predictive model^81^.

The gradient boosting regressor (GBR) is the standard form of this approach. It sequentially fits new trees to the negative gradient of the loss function, improving performance with each iteration. Therefore, the predicted values of the GBR model can be mathematically expressed as follows^81^:

18 $$T\left({x}_{i}\right)={\sum }_{k=1}^{K}{f}_{k}\left({x}_{i}\right)$$

where ${f}_{k}\left({x}_{i}\right)$ is the output of the *k*th tree and $T\left({x}_{i}\right)$ is the predicted value of the GBR model. While flexible and relatively straightforward, it can be slower to train and may require careful hyperparameter tuning^81^.

Extreme gradient boosting (XGBoost) refines gradient boosting by optimizing tree construction and enabling parallelization. It employs techniques such as shrinkage (learning rate), column subsampling, and regularization (through L1/L2 penalties) to mitigate overfitting^82^. Additionally, the XGBoost uses a more advanced tree splitting algorithm and supports out-of-core computing for large datasets^82,83^.

The Light Gradient Boosting Machine (LightGBM) also builds on gradient boosting but focuses on speed and memory efficiency. It uses a histogram-based algorithm that groups continuous features into discrete bins, reducing the complexity of splitting. This approach, coupled with a technique called gradient-based one-sided sampling, enables LightGBM to handle large-scale data with less computational overhead, sometimes offering faster training compared to XGBoost while maintaining competitive accuracy^84^.

Categorical boosting (CatBoost) focuses on handling categorical features within the data^85^. It automatically encodes such data without extensive preprocessing and uses ordered boosting, where each tree is trained on a random permutation of the data to reduce prediction bias^86^. Because it tackles categorical variables natively, CatBoost often avoids the pitfalls of one-hot encoding or other feature engineering steps^87^, making it an appealing choice for datasets with many discrete features^86,88^.

Histogram-based gradient boosting (also known as HistBoost) approach, like LightGBM, bins continuous variables to accelerate splitting decisions^89^. By discretizing continuous values into buckets, it speeds up training on large datasets but might introduce minor accuracy trade-offs due to the binning approximation^90,91^.

Adaptive boosting (AdaBoost) also combines multiple weak learners, but it does so differently. Instead of fitting new models to the gradient of the loss, it adjusts the weights of incorrectly classified samples so that subsequent learners focus more on challenging examples^92–94^. Though often implemented with decision trees, AdaBoost can use other base learners, making it versatile. However, it can be sensitive to noisy data, and its performance can degrade if there is a notable level of noise^95^.

These boosting methods share the fundamental idea of ensemble learning but differ in optimization techniques, handling of continuous or categorical data, speed, and complexity of implementation. A more comprehensive description of the methods can be found in the references mentioned.

### Random Forest (RF) and Extra Tree

Random Forest (RF) and Extra Tree are ensemble learning techniques that build multiple decision trees using different subsamples of the training data and a subset of features. In Random Forest, each tree grows to the maximum depth without pruning, and its predictions are combined by averaging for regression or majority voting for classification^96^. Because these trees are trained in parallel and rely on slightly different subsets of data and features, they tend to exhibit low correlation^97,98^. As a result, RF offers good predictive accuracy and robust performance against overfitting^99^.

Extra Trees (also known as extremely randomized trees) follow a concept like RF but introduce even more randomness^100^. In Extra Trees, split thresholds are chosen at random, not just the subset of features^101^. This randomness can produce more diverse trees in the ensemble, potentially improving performance in certain cases. However, depending on the dataset, Extra Trees may be more sensitive to hyperparameters like the number of features considered at each split^102,103^.

While both RF and Extra Trees are ensemble techniques, they differ distinctly from boosting techniques. In boosting, models are added sequentially, with each new learner focusing on the errors of its predecessors. This process, guided by a stepwise correction of the ensemble’s residuals (from actual values), can yield high accuracy but may require careful parameter tuning and longer training times^104^. By contrast, RF and Extra Trees train all trees in parallel, reducing the overall computational load. They also balance variance and bias differently, relying more on averaging unpruned, highly varied trees rather than incrementally refining residuals. This parallelism often makes RF and Extra Trees faster to train and easier to tune, though they may not always achieve the same level of performance that well-tuned boosting methods can provide^98,100,103^. Additional explanations of the techniques described are included in the referenced works.

### Support vector regressor (SVR)

SVR is a type of AI algorithm based on support vector machines (SVM), designed for regression tasks. The architecture of SVR is built around the concept of finding a hyperplane (or decision boundary) in a high-dimensional space that best fits the data points, with a focus on minimizing the prediction error while maintaining simplicity in the model^105^. Unlike other regression models that minimize the prediction error directly, SVR introduces the concept of an “epsilon-tube,” which defines a margin of tolerance within which predictions are considered acceptable^106^. The aim of SVR is to find a hyperplane that fits as many points as possible within this epsilon margin, while minimizing the model’s complexity. The objective can mathematically be written as follows^107,108^:

19 $${{Obj}}:\left(\frac{1}{2}\right){||w||}^{2}+C{\sum }_{i=1}^{n}({\xi }_{i}+{\xi }_{i}^{\ast })$$

20 $$\begin{array}{rcl}{{Subject}}\,{{to}}\left\{\begin{array}{c}{y}_{i}-(w\cdot {x}_{i}+b)\le \varepsilon +{\xi }_{i}\\ (w\cdot {x}_{i}+b)-{y}_{i}\le \varepsilon +{\xi }_{i}^{\ast }\\ {\xi }_{i},{\xi }_{i}^{\ast }\ge 0\end{array}\right.\end{array}$$

where $w$ is the weight factor, $b$ is bias term, and ${\xi }_{i}$ and ${\xi }_{i}^{* }$ are slack variables represent the deviations of the data points outside the epsilon-insensitive tube, $\varepsilon$ is loss function parameter, which defines the width of the tube around the regression function and $C$ is the regularization parameter. The referenced works provide additional details on how the method works and should be implemented in the work.

### Gaussian process regression (GPR)

GPR is a powerful, non-parametric Bayesian approach to regression tasks that models the relationship between input data and outputs using probability distributions over functions. The core idea behind GPR is to assume that the data points are drawn from a multivariate Gaussian distribution, where any subset of these points will also follow a Gaussian distribution. Rather than directly learning a function to map inputs to outputs, GPR defines a prior over possible functions and then updates this prior with observed data to create a posterior distribution^109^. The goal of GPR is to make predictions by estimating the mean and variance of this posterior, providing not only the predicted value but also a measure of uncertainty in the prediction^109,110^. The method discussed is elaborated in greater detail in the sources cited.

### K-Nearest neighbors (KNN)

KNN is a simple yet powerful machine learning algorithm used for both classification and regression tasks. The key idea behind KNN is to make predictions based on the similarity of a new input to existing data points in the training set. It works by identifying the ‘*K*’ closest points (or neighbors) to a given query point^111^, using a distance metric such as Euclidean, Manhattan, or Minkowski distance. In regression tasks, KNN takes the average of the neighboring data points’ values to make a prediction^112^. Practically, KNN is a non-parametric and lazy learning algorithm, meaning that it makes no assumptions about the underlying distribution of the data and does not build a model during the training phase. Instead, it stores the entire training dataset and defers computation until prediction time^113,114^. When a new query point is introduced, the algorithm calculates the distances between this point and all training points. More comprehensive coverage of the method is available in the cited materials.

### Linear and non-linear models (non-AI)

Linear regression assumes a direct proportional relationship between input features and target variables, represented by a simple weighted sum of input features plus an intercept. While interpretable and computationally efficient, linear models struggle with non-linearity^115^. Non-linear models extend this capability by considering higher orders of the independent variables to get closer to the training data. These models can capture complex dependencies at the cost of increased computation and reduced interpretability^116^.

### Index and evaluation metrics

Once the AI models are trained by using the explained techniques, their performance needs to be analyzed using insightful metrics. In this work, five different performance indexes are used which have been listed below along with their mathematical formulas^117,118^:

21 $${R}^{2}=1-\frac{{\sum }_{i=1}^{n}{\left({y}_{i}-\hat{{y}_{i}}\right)}^{2}}{{\sum }_{i=1}^{n}{\left({y}_{i}-\bar{y}\right)}^{2}}$$

22 $${{{Residual}}\,{{Value}}\,({{RV}})=1-R}^{2}$$

23 $${{Absolute}}\,{{Error}}={|}{y}_{i}-\widehat{{y}_{i}}{|}$$

24 $${{Mean}}\,{{Absolute}}\,{{Error}}\,({{MAE}})=\frac{1}{n}{\sum }_{i=1}^{n}{|}{y}_{i}-\widehat{{y}_{i}}{|}$$

25 $${{Root}}\,{{Mean}}\,{{Squared}}\,{{Error}}\,({{RMSE}})=\sqrt{\frac{1}{n}{\sum }_{i=1}^{n}({{y}_{i}-\widehat{{y}_{i}}})^{2}}$$

26 $${{Mean}}\,{{Absolute}}\,{{Relative}}\,{{Error}}\,({{MARE}})=\frac{1}{n}{\sum }_{i=1}^{n}\left|\frac{{y}_{i}-\widehat{{y}_{i}}}{{y}_{i}}\right| \times 100$$

where in these equations ${y}_{i}$, $\hat{{y}_{i}}$, and $\bar{y}$ are actual, predicted, and mean of the actual values, respectively and $n$ is the number of datapoints.

## Results and discussion

### Optimization of hyperparameters of the AI models

Hyperparameters are variables that define the AI models’ structure or how it is trained, but they are not learned during the training process and need to be set before the training process starts. Hyperparameter optimization aims to find the best combination of these values to maximize model performance.

In this paper, a systematic approach was used to optimize hyperparameters in which hyperparameter combinations within predefined values are searched, and the performance of the model over each combination was evaluated. Then, for continuous hyperparameters, when the performance vs. hyperparameter plot saturates, the point is chosen as the best value for the hyperparameters. For non-quantitative values (e.g., Kernel selection for the SVR model), the one with the highest performance was chosen. Also, to ensure that the models are not overfitted, for each step of the hyperparameter searching a *k*-fold cross-validation technique^119,120^ was used with a *k* = 5. More details about the process of optimization, including the searching range and the optimum values, have been provided in Table S2 in the supplementary information to support the reproducibility of the results of this paper. Taking the CFNN model as an example and the superior model in terms of predictive accuracy, the results of its hyperparameter optimization are provided in Table S3 in the supplementary information. Looking at this table, with a network containing 5 hidden layers and the size of the layers of [10 30 30 30 30], CFNN has resulted in the best performance across the searching range. It should be noted that no predefined specific structure (e.g., symmetrical or neat) among the network sizes was set for the process, and the best network size was only considered according to its performance metrics. Also, as the CFNN model takes a long time for the training process, a step of 5 was considered for the size of each hidden layer to reduce the computational burden of this process.

As was mentioned in earlier sections, in this study, the potential of using AI techniques for predicting time-independent (average) and time-dependent (dynamic) loss has been shown. In the following, first, the results of the simpler yet useful AI models that predict average losses have been provided. The output of these models will only depend on the configuration of the motor, and the instance of time does not affect the predicted AC loss. Then, the dynamic model that predicts the exact AC loss for any instance of time within a working cycle is proposed.

### Average AC loss prediction: static estimation

Table 1 provides the performance of the best model of each of the 14 AI models and 2 mathematical fittings that have been developed in this work. Looking into the details of the table, the CFNN model has the highest *R*-squared of 99.994% and the lowest error metrics (RV of 5.99E−05, RMSE of 4.135, and MARE of 0.316%), outperforming all models in accuracy of predictions. In terms of the prediction speed, it also demonstrates a fast prediction, where it takes 46 ms to predict the AC loss for more than 2800 different configurations of motor, making it the best performing model overall. As an alternative, FFNN follows closely in accuracy but is slightly less precise (*R*-squared: 99.987%) while offering a faster estimation time of 29 ms.

**Table 1:** Benchmarking of the performance of different AI and non-AI algorithms for the prediction of temporally independent AC losses

Algorithm	*R*-squared (%)	RV	RMSE	MARE (%)	Testing time for estimation of whole data [ms]
CFNN	99.994	5.99E−05	4.135	0.316	46
FFNN	99.987	1.33E−04	5.804	0.418	29
SVR	99.966	3.36E−04	7.981	0.502	173
Catboost	99.944	5.60E−04	9.241	0.610	67
XGBoost	99.896	1.04E−03	12.503	0.881	21
LightGBM	99.890	1.10E−03	15.153	0.896	342
GPR	99.903	9.75E−04	16.230	1.018	19
GBR	99.880	1.20E−03	17.201	1.159	98
HistBoost	99.857	1.43E−03	18.855	1.364	296
Random Forest	99.806	1.94E−03	21.376	1.172	491
Extra tree	99.690	3.10E−03	27.802	1.442	518
KNN	98.689	1.31E−02	59.527	3.661	49
Non-linear	98.395	1.61E−02	65.861	4.895	14
GRNN	97.229	2.77E−02	90.640	4.748	76
Linear	91.546	8.45E−02	151.141	11.160	10
Adaboost	91.229	8.77E−02	153.941	16.860	326

Comparing the CFNN model’s accuracy with the non-AI models shows clear superiority across all reported metrics. Its coefficient of determination (99.994%) shows that almost all variability in the target variable is captured, whereas classical linear regression explains only 91.546% and the more flexible but still hand-crafted non-linear fit reaches 98.395%. Similarly, error measures also confirm this advantage. CFNN lowers the RMSE to 4.135, compared with 151.141 for the linear model and 65.861 for the non-linear model, a reduction of roughly 97% and 94%, respectively. The MARE shows the same, dropping from double-digit percentages (11.160% for linear, 4.895% for non-linear) to only 0.316% for CFNN. Looking at the differences in response times, non-AI fits generate faster responses due to the simplicity of the model and the equations. CFNN evaluates the entire test set in 46 ms, which is only a few tens of milliseconds slower than non-AI models (10–14 ms). However, while the non-AI methods require minimal runtime to make predictions and are relatively advantageous over this factor, the CFNN’s response time is still comfortably inside real-time limits. That said, their simplicity in the model development process makes them still a convenient and valuable tool that can be easily implemented. This highlights how AI techniques can improve the performance of surrogate models for critical applications like aviation.

**Fig. 5:**
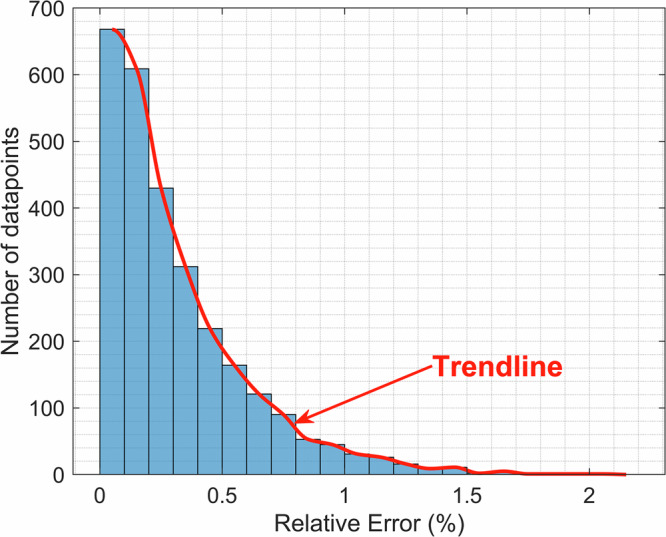
Distribution of the relative error of the CFNN model for the whole dataset.

Taking a deeper look into the CFNN model results, Fig. 5 shows that the CFNN model can estimate the AC loss of 96.27% of the total datapoints (2816 different motor configurations) with a relative error (RE) of <1%. Also, there are no points that exceed 2.27% RE, showcasing the robustness of the developed model for the AC loss estimation of the superconducting propulsion motor.

**Fig. 6:**
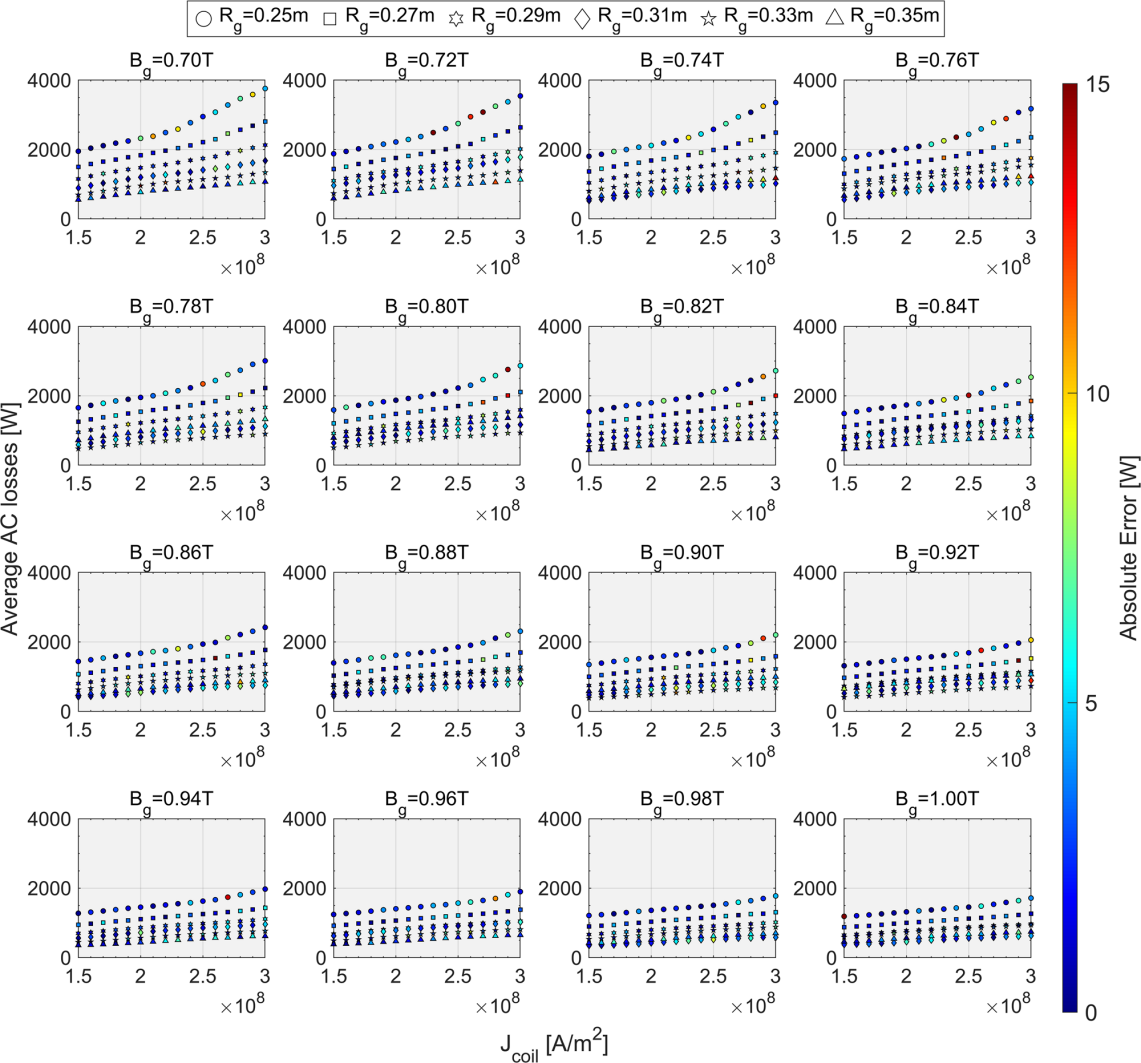
Visualization of the pattern of the average AC loss with variation of the input parameters of the model.

Figure 6 also provides a full, detailed visualization of the pattern of AC loss with respect to the changes of input parameters. As is evident from this figure, decreasing *B*_g_ and *R*_g_, and increasing *J*_coil_ will generally increase the total AC loss of the machine. Also, looking at the difference between the plots in Fig. 6, increasing the magnetic field plays a crucial role in decreasing the AC loss.

### Temporally dependent AC loss prediction: dynamic prediction

Following the presentation of various static AC loss predictor AI models in the previous section and the subsequent benchmarking study that identified the CFNN as the AI model with the highest overall accuracy for loss prediction, this section extends the analysis to dynamic (time-dependent) AC loss behavior. Whereas the static models capture total losses under a steady operating condition, the time-dependent approach provides an instant-by-instant estimation of AC loss over transient or periodic cycles. This capability is especially relevant for real-time monitoring, control, and design optimization, where knowledge of how losses evolve over the course of each electrical cycle can influence motor reliability, thermal management, and operational decisions. In other words, with accurate predictions of transient and periodic AC loss, researchers are ultimately enabled to effectively use these surrogate models in system-level models where the inputs and outputs of the model can affect the operation of other parts of the system.

Using the time-dependent dataset that was described in the section “Methods”, the dynamic CFNN model was trained with 80% of the available data, which were chosen randomly to avoid any bias in the selection process, and then tested with 20% of the remaining data. The same optimization attempts for the static models that were described previously were used to ensure the model shows its utmost capabilities. The results of the grid search showed that the CFNN model with an architecture of 3 hidden layers and neuron configuration of [15 15 15] performs the best. The results of the tests show that the CFNN model can predict dynamic AC losses with an *R*-squared of 99.973%, RMSE of 17.66, and MARE of 2.79%.

**Fig. 7:**
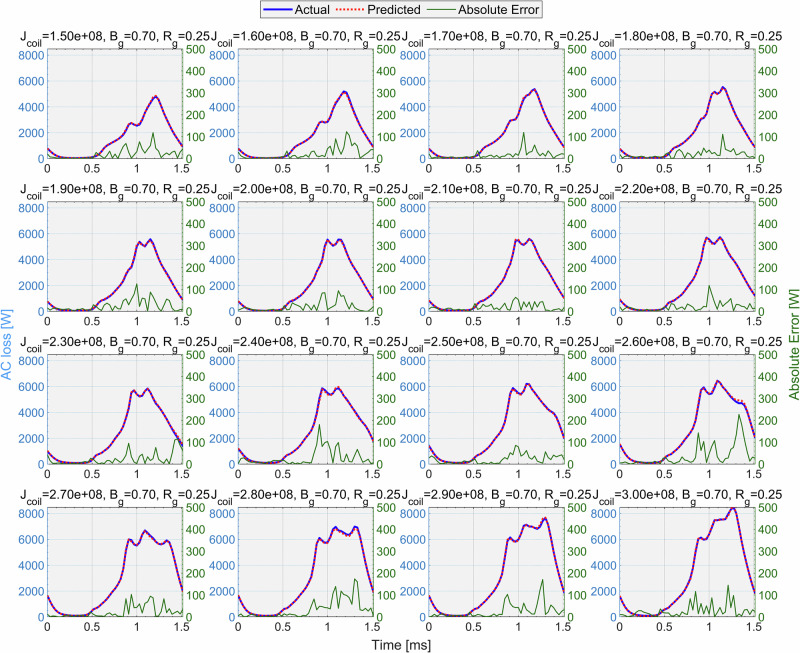
Comparison of the time-dependent AC loss of the motor with different *J*_coil_ and the predicted morphology of the CFNN model.

Figure 7 showcases the evolution of AC loss over half an electrical cycle under different current densities (*J*_coil_). The blue curves represent reference data generated from detailed *T*–*A* formulation electromagnetic simulations of the superconducting motor’s slot model (which was explained in the section “Methods”), while the red curves represent CFNN predictions. The green curve also shows the difference between predicted and actual values. Examining the figures, as *J*_coil_ increases, the peak loss is amplified, and its phase is slightly shifted. The CFNN demonstrates remarkably close agreement in both amplitude and timing of these peaks, which is evidence for CFNN’s ability to learn the morphological features of the waveform rather than simply interpolating magnitudes.

Similarly, Figs. S4 and S5 in the supplementary information compare actual and predicted temporal-dependent AC losses under varying magnetic flux amplitude (*B*_g_) and air-gap radii (*R*_g_), respectively. Changes in the external field strength modify the loss waveform’s amplitude and can alter the time at which peak losses occur. Also, changes in *R*_g_ substantially change the waveform pattern of the AC loss by affecting the magnetic field distribution around the superconducting coils. Despite these shifts, the CFNN’s predictions closely follow the real data, evidencing that the model takes on not just the peak height but also details in how the waveform bumps up and down.

Even though small discrepancies appear, particularly around sharp gradients or abrupt changes, they remain within acceptable bounds for engineering analyses in aerospace applications. The fact that the CFNN handles these variations smoothly makes it a compelling surrogate model for rapidly predicting AC losses without the computational overhead of running a full electromagnetic FE simulation each time there is interest in a modified design parameter. These results show that the application of AI models for the estimation of losses is not bound to average loss values, and advanced intelligent techniques like CFNN can offer valuable dynamic insights into losses with very high accuracy.

For the sake of comparison of the results of the CFNN model with the least accurate technique and also non-AI models, the same approach was also implemented for the linear regression technique, and the results are presented as Figs. S6–S8 in the supplementary information.

### Insights into the generalizability of the model

#### Influence of random selection of data

In the process of developing predictive AI models, the randomness inherent in splitting data into training and testing sets can introduce small variations in model performance. Therefore, to investigate the consistency and stability of our approach and the impact of random train-test splits on the model’s accuracy, the CFNN model was trained 100 times from scratch, each run with a distinct random seed. On average, each training process took around 2 h. This approach allowed observation of variability in performance due to different random selections of training and testing subsets at each try.

Figure S9 in the supplementary information shows how the performance of the model changes over these runs. Looking at the left figure, which is for the dynamic model, an average *R*^2^ of 99.975% and MAE of 10.27 W was achieved across these runs, closely aligning with the above values shown in the previous section, thereby indicating high consistency and stability of the model’s performance. Additionally, the standard deviation computed across the 100 repetitions was found to be 3.79e−05 for *R*^2^ and 1.02 W for MAE, demonstrating minimal variation between the individual results. Similarly, for the right figure, which corresponds to the static model, an average *R*^2^ of 99.987% and MAE of 4.05 W was achieved across the 100 repetitions. The evaluated standard deviation across the runs was 4.13e−05 for *R*^2^ and 0.58 W for MAE, confirming the model’s robustness and stability with minimal variability in results.

Such minimal variance and proximity of the average accuracy to established benchmarks underscore the robustness and reliability of the model, highlighting its strong generalizability to unseen data. These outcomes confirm that the model’s predictive capabilities remain consistently high, regardless of changes introduced by different random train-test partitions. This proves the suitability of the developed models in the previous section for use in broader motor configurations.

### Capability of extrapolating for out-of-the-box predictions

The AI models are mainly designed for interpolation tasks, where the predictions are made within the trained range. The training range that was discussed in the section “Methods” and summarized already satisfies the expected range of parameters in which the model is used in the system-level model defined by Airbus. Going beyond the requirements of the project, we test the CFNN model for the prediction of conditions where the inputs are out of the training range, known as extrapolation. In other words, we test the extrapolative performance of the CFNN model when it is used in a system-level model in Simulink and encounters motor configurations that are out of the bounds for which it was trained.

To do so, a new dataset containing 1059 new motor configurations was constructed. These new data were generated using the same COMSOL model that was presented in the section “Methods”, but with the new range for input parameters, which results in a motor configuration that does not seat within the bounds of the training set for CFNN models. These new ranges have been summarized in Table S4 in the supplementary information. In practice, to generate the new data, the lower bound of *B*_g_ decreased from 0.7 in the initial data to 0.6, and the upper bound was moved from 1 to 1.1 T. Also, the *J*_coil_ bounds were changed from 1.5e8 to 1.3e8 and from 3e8 to 3.2e8 A/m^2^ for lower and upper bounds, respectively. Figure S10 is also provided along with Table S4 in the supplementary information to visually show the new motor configurations in comparison to the trained data.

Using the same dynamic and static models CFNN model that was discussed in previous sections, the AC losses of new motor configurations are predicted and compared with the actual results. For the static CFNN model, the model demonstrates a performance of 97.31% *R*-squared, MARE of 7.22%, and RMSE of 143.49 on unseen motor configurations by network. In terms of the dynamic CFNN model, the model indicated a great performance of 99.54% *R*-squared, 4.59% of MARE, and 113.61 RMSE.

**Fig. 8:**
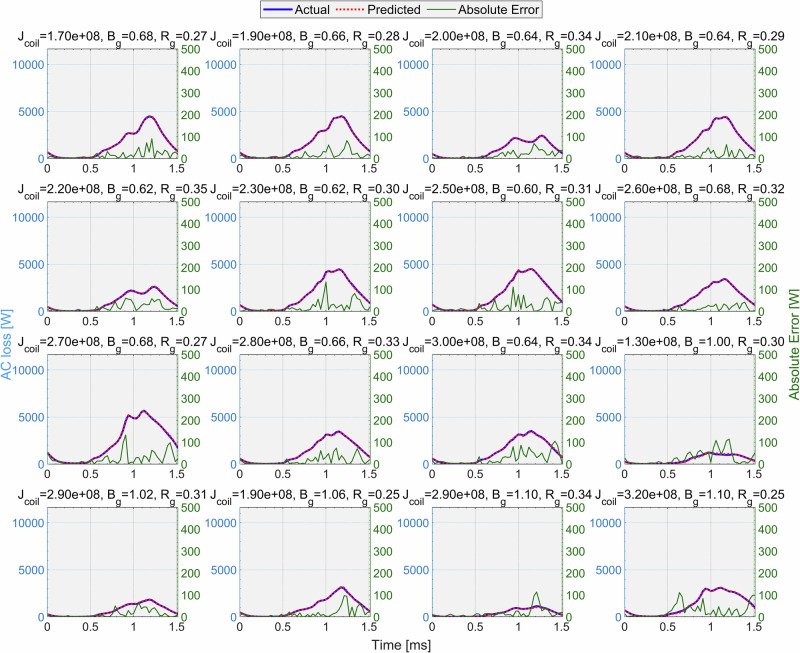
Visualization of the actual AC loss value vs. the predicted value of CFNN in the extrapolating task.

In Fig. 8, several randomly selected cases out of 1059 new cases are plotted to demonstrate the model’s performance over these new motor configurations. These plots demonstrate that the absolute error traces remain relatively small in most cases, particularly outside the main peak regions, suggesting that the predictions are accurate over a broad portion of each time series. Although the model was not trained on these motor configurations, it has accurately predicted the morphology of the AC loss waveforms. This indicates that even though the CFNN model is a black-box AI, whose internal mechanisms for capturing the underlying physics are not explicitly explainable, it has effectively learned the patterns and physics of AC losses during training. Consequently, it not only performs well within the bounds of the initial dataset but also yields relatively accurate predictions for motor configurations beyond that range.

### Influence of data sampling and limitations of data

It is a common misconception in artificial intelligence (AI) research that expanding the training dataset inevitably results in enhanced model accuracy and generalizability. However, an increase in data volume alone does not necessarily improve model performance. The effectiveness and reliability of an AI model depend primarily on the representativeness and quality of the training data rather than merely the quantity.

For a model to effectively capture and predict physical behaviors, the data utilized during training must comprehensively encapsulate the underlying patterns, trends, and variations observed within the specific operational context where the model will ultimately be applied. If the data fails to reflect these physical behaviors accurately, the resulting AI model may struggle to generalize and exhibit poor predictive capabilities when introduced to real-world conditions or scenarios beyond the scope of its training examples. Therefore, careful attention should be devoted to ensuring that the dataset covers the relevant range of physical phenomena and adequately represents the variability of practical situations.

As was seen in Figs. 7, S4, and S5, the pattern of the AC loss waveforms substantially changes when working conditions (*J*_coil_ and *B*_g_) or motor design changes (*R*_g_) occur due to alterations in the hysteresis and magnetization losses in the motor. Therefore, the input data for the model needs to be carefully analyzed to determine whether it represents these physical phenomena to ensure the final model outputs reliable predictions. To do so, an analysis of the limitations of the data and their effect on the performance of the model has been conducted. Focusing solely on the CFNN, identified as the superior technique in previous sections, different randomly selected proportions of the dataset were considered for the training process, and the models’ performance was tested using the remaining data. Figure S11 shows how the performance of the model changes when the training set size changes. These plots are also known as learning curves. As it appears in Fig. S11 in the supplementary information, for the static CFNN model (right side), the model’s accuracy does not change significantly for training sets bigger than one-quarter. This means that only 700 motor configurations were enough to represent the physical characteristics of AC losses for the studied motor and within the defined range of parameters. For the dynamic model, however, the threshold is about 50% of the data, where for larger sets the accuracy of the model seems to be stabilized.

### SIMULINK implementation

**Fig. 9:**
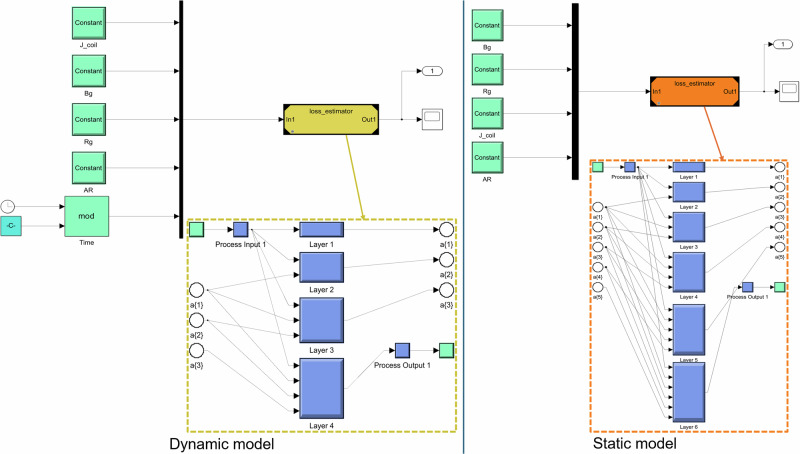
Implementation of CFNN models in the Simulink environment. Dynamic model (left) and static one (right).

After completing the performance analysis, the models are ready for implementation to simulate AC losses in various motor configurations. To enable this, the trained model is saved as a separate source file, which can later be transferred to the required hardware and imported into the relevant code. The model then receives four input values (*R*_g_, *B*_g_, *J*_coil_, and AR) for the static model, or five input values (*R*_g_, *B*_g_, *J*_coil_, AR, and time) for the dynamic model, to predict the AC losses. As the system-level model is developed in the Simulink environment, the complex CFNN model is imported, masked, and configured to receive four or five input parameters (depending on whether the static or dynamic model is used) to predict the AC losses. Figure 9 illustrates a straightforward example of these models implemented in Simulink, where these inputs are provided to the model using constant or signal blocks, and the model’s output is then connected to other components of the system-level simulation.

Since the dynamic model has been trained to predict AC losses for half-cycle intervals, the values obtained from the clock signal require pre-processing before inputting them into the model. Specifically, the clock time is divided by 0.001515, representing the duration of a half-cycle, and the remainder from this division is then used as the input for the loss estimator model. To evaluate the models’ performance within Simulink, simulation durations of 1 and 5 s were chosen, with a step size set at 1 µs. The total simulation times are summarized in Table S5 in the supplementary information using the normal solver with a fixed time-step. The results indicate that the dynamic model operates significantly faster than the static model. This performance difference arises from the complexity of the CFNN model architecture, which increases with each additional layer added to the network. As detailed earlier, the optimal CFNN configurations identified for the static and dynamic models are [10 30 30 30 30] and [15 15 15], respectively. In other words, the static model has 5 hidden layers while the dynamic model has only 3 layers. Given that the static model contains more layers and neurons, it requires additional computational time to generate predictions for each Simulink simulation time-step. This underscores the importance of balancing model precision against response time when deploying AI models in practical applications.

### Discussion and future implications of the proposed and developed models

Since advanced powertrain and propulsion designs often rely on system-level modeling, an accurate yet computationally light surrogate model for superconducting motor losses is vital. According to the results that were shown in these sections, CFNN’s time-dependent approach offers several benefits:

- *Thermal management and feasibility checks*: By accurately predicting AC loss patterns for various motor configurations—which, in fact, represent the heat loads on the motor’s cooling system—the model can assess the feasibility of a design for use in aircraft. This is done by considering the constraints of the onboard cooling system and ensuring that the superconducting coils are maintained within safe temperature limits.
- *Accelerated design cycles*: Rather than relying exclusively on full electromagnetic FEA simulations, often computationally expensive, the CFNN allows designers to run thousands of parametric sweeps in a fraction of the time. This accelerated process is particularly beneficial during early-stage conceptual design of novel superconducting motors, where trade-offs between geometry, material choice, and operational margins must be evaluated quickly. Although there is an upfront time and computational cost of producing the training data, this can be done early in the design cycle before major engineering commitments have taken place and can be largely automated. The availability of trained models for quickly available but accurate trade studies holds great potential and goes far beyond only AC loss estimation, but the effective use of these methods requires some adjustment to the normal development workflow.
- *Reliability and lifetime estimation*: Accurate AC loss prediction, especially when combined with thermal and mechanical stress models, provides a more faithful representation of real-world operating conditions over the motor’s entire duty cycle. This fidelity informs maintenance schedules and helps avoid overdesign, in turn saving weight—an especially critical factor in aviation.
- *Real-time monitoring*: By deploying the CFNN in a hardware-accelerated environment (e.g., a GPU), the system can achieve near-instantaneous predictions of dynamic AC losses. If the motor experiences sudden load changes—common during take-off, climb, or turbulence—the CFNN model can be used to provide inputs for control systems to help them react quickly and effectively to mitigate any risk of thermal overrun or inefficiency.

## Conclusion

Superconducting motors, with their high electrical efficiency and lightweight design, enable future electric aircraft to benefit from a propulsion system with a high specific power density. However, these advantages are subject to maintaining cryogenic temperatures. Therefore, any heat generated within the system must be effectively dissipated, which comes at the cost of additional weight due to bulky cooling systems and coolant fluids. As a result, the AC losses of the motor, under various design parameters and operating conditions, must be analyzed within a holistic system model.

This paper, by developing artificial intelligence (AI) surrogate models for an HTS superconducting motor, demonstrates the potential of intelligent techniques for accurately estimating AC losses as part of a digital twin framework. Furthermore, this study advances the understanding of how deep learning techniques can capture the time-dependent patterns of AC losses in the motor under different design and operating conditions using a cascade-forward neural network (CFNN). The results show that the CFNN model, by learning the morphology of AC loss waveforms, can predict AC loss patterns across all design and operating conditions within the trained range. Additional findings of this study include the following:

- The CFNN technique outperformed all 15 other AI and non-AI regression techniques that were employed in the benchmarking study of this paper in terms of the accuracy of the prediction of AC losses, with an *R*-squared of 99.994%.
- More than 96% of the datapoints had <1% relative error compared with their finite element model values and were predicted by the CFNN static model.
- The CFNN model has shown very high accuracy of prediction of time-dependent AC losses with an *R*-squared of 99.973%.
- The required time of the dynamic model for estimation of losses is less than 9 ms in the developed test bed. This can further be decreased by using near-real-time fast-computation techniques.
- A bigger dataset with more data does not necessarily lead to a more generalizable AI model. Therefore, careful attention needs to be paid when the raw data is collected/constructed for the development of AI models.
- The developed CFNN model is mainly developed to accurately predict AC losses within the trained range, but it can still be used for the motor configurations that are out of the training range in the initial dataset.

## Supplementary information

Supplementary materials

## Data Availability

All data generated or analyzed during this study are included in this published article (and its Supplementary Information files).

## Code availability

The project codes cannot be directly shared due to project confidentiality restrictions. However, the model development process is clearly illustrated in Fig. S1 in detail, and all parameter values are provided in Table S2, in the supplementary files. Furthermore, supplementary information contains both a general algorithm for developing all models (Algorithm S1) and a detailed algorithm for the CFNN model (Algorithm S2), as the best technique identified for the problem in this paper.
