# Supplementary material for: Advanced deep-learning model for temporal-dependent prediction of dynamic behavior of AC losses in superconducting propulsion motors for hydrogen-powered cryo-electric aircraft
Source: Commun Eng. 2025 Dec 17;4:221. doi: 10.1038/s44172-025-00554-8 (PMC12738581; doi:10.1038/s44172-025-00554-8)
Supplement: Supplementary file 1 — Supplementary materials [file 44172_2025_554_MOESM1_ESM.docx]

## Supplementary information: **Deep-learning model for temporal-dependent prediction of dynamic behavior of AC losses in superconducting propulsion motors for hydrogen-powered cryo-electric aircraft**

Shahin Alipour Bonab^1^, Frederick Berg^2^, Wenjuan Song^1^, Alexandre Colle^3^, and Mohammad Yazdani-Asrami^1,*^

^1^ CryoElectric Research Lab, Propulsion, Electrification & Superconductivity Group, Autonomous Systems and Connectivity Division, James Watt School of Engineering, University of Glasgow, Glasgow, G12 8QQ, United Kingdom

^2^ Airbus X-Labs, Willy-Messerschmitt-Straße 1, 82024 Taufkirchen, Germany

^3^ Airbus UpNext, Toulouse, France

^*^Corresponding author’s email address: mohammad.yazdani-asrami@glasgow.ac.uk


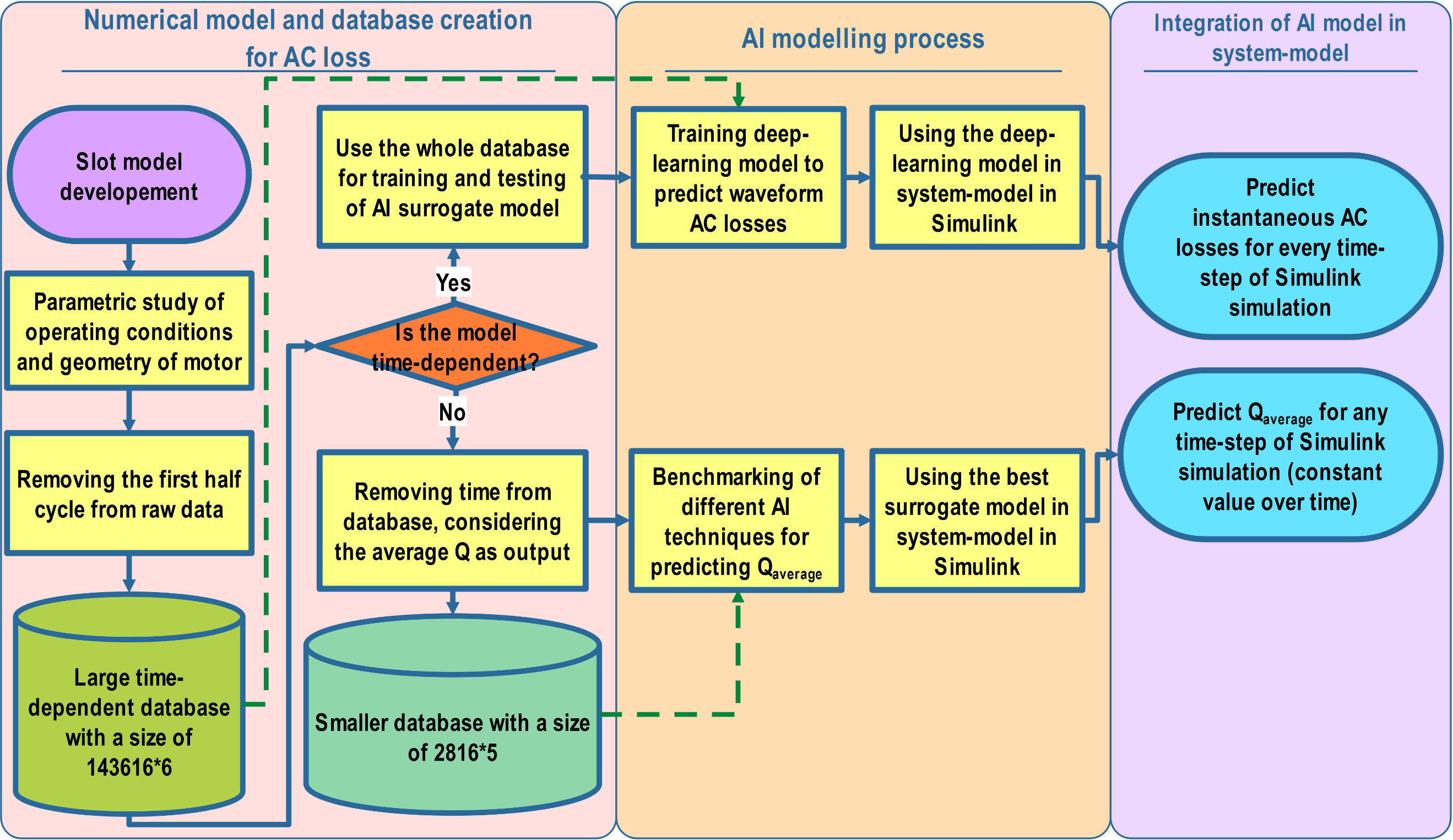


Figure S1. Flowchart of the process of developing time-dependent and time-independent AI surrogate models

This figure illustrates the workflow of this paper’s research.

| **(a)**  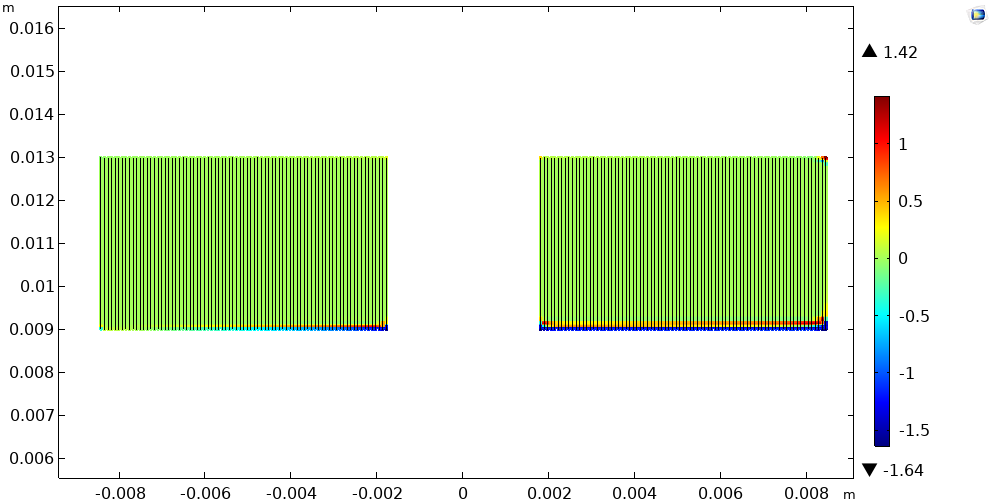 | **(b)**  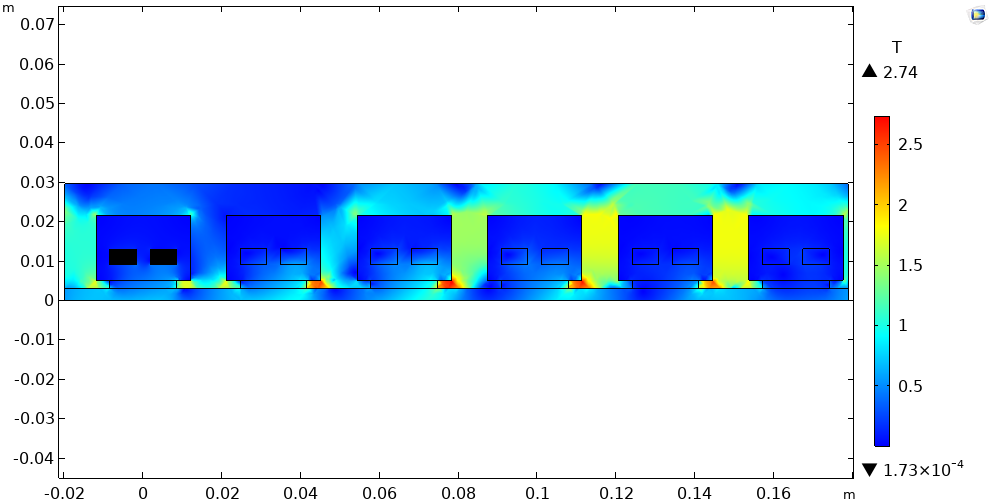 |
| --- | --- |

Figure S2. A sample of the distribution of J/Jc (a) and magnetic flux intensity (b) used during the numerical simulation.

This figure shows how the magnetic field within the motor geometry and electrical field, and normalized J (J/J_c_) within the superconducting parts are distributed. These contours were directly captured from the COMSOL model at the instance of three-quarters of the working cycle for the motor configuration of AR=0.379, R_g_=0.25m, B_g_=0.7T, and J_coil_=1.5e8 A/m^2^.


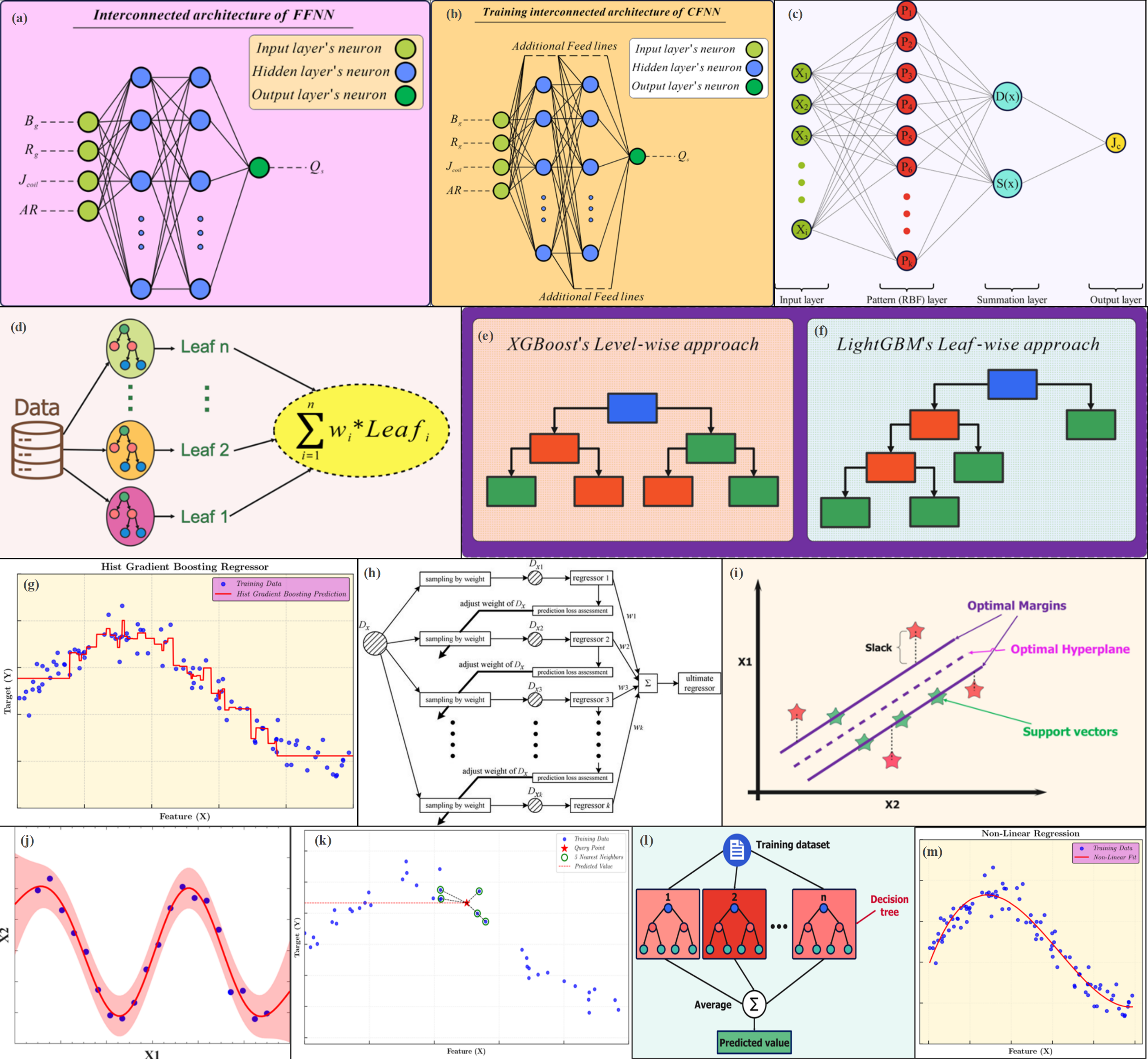


Figure S3. Schematic illustrations of different AI techniques used in this work. From top-left to bottom-right the illustrations show the architecture of (a) FFNN, (b) CFNN, (c) GRNN, (d) GBR, (e) XGBoost, (f) LightGBM, (g) Hist-boosting, (h) Adaboost, (i) SVR, (j) GPR, (k) KNN, (l) Random-forest and Extra tree, (m) Non-linear regression

This figure provides a visual presentation of how each AI technique that is used in the paper works.

Table S1. Range of the design and operating parameters

| Parameter | B_g_ [T] | R_g_ [m] | J_coil_ [A/m^2^] | AR | Static Q_sc_ [W] | Dynamic Q_sc_ [W] |
| --- | --- | --- | --- | --- | --- | --- |
| Range | 0.7 to 1 | 0.25 to 0.35 | 1.5E8 to 3E8 | 0.30 to 0.3737 | 313.98 to 3757.34 | 6.56 to 8472.21 |

This table summarizes the range of each input parameter in FEM model in COMSOL and their resulting AC loss (both static and dynamic). Resulting database were used to develop AI models.

Table S2. The search range and the optimal value of hyperparameters of different AI and non-AI models for static models

| Algorithm | Hyperparameter | Search range | Optimum value | Hyperparameter | Search range | Optimum value |
| --- | --- | --- | --- | --- | --- | --- |
| CFNN | Layers | 1-5 | 5 | Neurons at each hidden layer | 5-40 | [10 30 30 30 30] |
| FFNN | Layers | 1-5 | 4 | Neurons at each hidden layer | 5-30 | [15 5 5 15] |
| SVR | c | 0.01 - 1000 | 1 | degree | 2-6 | 2 |
|  | epsilon | 0.0001-1 | 0.01 | gamma | scale, auto | scale |
|  | kernel | linear, poly, rbf | rbf |  |  |  |
| CatBoost | depth | 4-8 | 6 | L2_leaf_reg | 1-7 | 5 |
|  | learning_rate | 0.01-0.2 | 0.1 |  |  |  |
| XGBoost | n_estimators | 10-1600 | 160 | subsample | 0.6-1.1 | 0.60 |
|  | max_depth | 1-14 | 4 | colsample_bytree | 0.6-1.1 | 1 |
|  | learning_rate | 0.05-0.95 | 0.1 |  |  |  |
| LightGBM | num_leaves | 10-400 | 50 | n_estimators | 10-5000 | 210 |
|  | learning_rate | 0.05-0.21 | 0.05 | min_child_samples | 0-10 | 6 |
| GPR | alpha | 0.000001-0.01 | 0.0001 | kernel | rbf, matern, rationalquadratic | rationalquadratic |
| GBR | n_estimators | 50-1500 | 250 | min_samples_split | 5-40 | 10 |
|  | max_depth | 3-9 | 3 | min_samples_leaf | 1-16 | 7 |
| HistBoost | max_depth | 3-11 | 3 | max_bins | 250-520 | 255 |
|  | min_samples_leaf | 5-100 | 7 |  |  |  |
| RF | n_estimators | 10-1000 | 170 | min_samples_split | 2-10 | 2 |
|  | max_depth | 2-30 | 5 | min_samples_leaf | 2-10 | 3 |
| Extra trees | n_estimators | 50-500 | 110 | min_samples_split | 2-50 | 4 |
|  | max_depth | 5-200 | 8 | min_samples_leaf | 2-50 | 2 |
| KNN | n_neighbors | 2-11 | 3 | p | 1 (Manhattan), 2 (Euclidean) | 1 |
|  | weight | uniform, distance | distance |  |  |  |
| GRNN | spread of RBF | 0.001-0.200 | 0.012 |  |  |  |
| Non-linear | degree | 2-10 | 3 |  |  |  |
| AdaBoost | n_estimators | 10-500 | 30 | learning_rate | 0.05-2 | 1.35 |
|  | loss | linear, square, exponential | exponential |  |  |  |

Table S3. Top 12 best network sizes (configurations) of CFNN model

| Network Size | RMSE | CV |
| --- | --- | --- |
| 10 30 30 30 30 | 4.135 | 5.99E-05 |
| 30 30 10 25 25 | 6.824 | 8.70E-05 |
| 20 15 30 25 25 | 8.862 | 8.00E-05 |
| 20 20 25 20 20 | 8.894 | 9.90E-05 |
| 5 25 25 25 30 | 9.034 | 9.00E-05 |
| 15 30 10 20 20 | 9.603 | 8.80E-05 |
| 30 30 25 15 25 | 10.339 | 1.05E-04 |
| 30 20 30 30 25 | 11.257 | 1.17E-04 |
| 5 25 40 35 25 | 11.853 | 1.22E-04 |
| 10 15 25 30 25 | 12.344 | 1.38E-04 |

This table provides the best 10 configurations of CFNN model during hyperparameter optimization, sorted with respect to RMSE values.


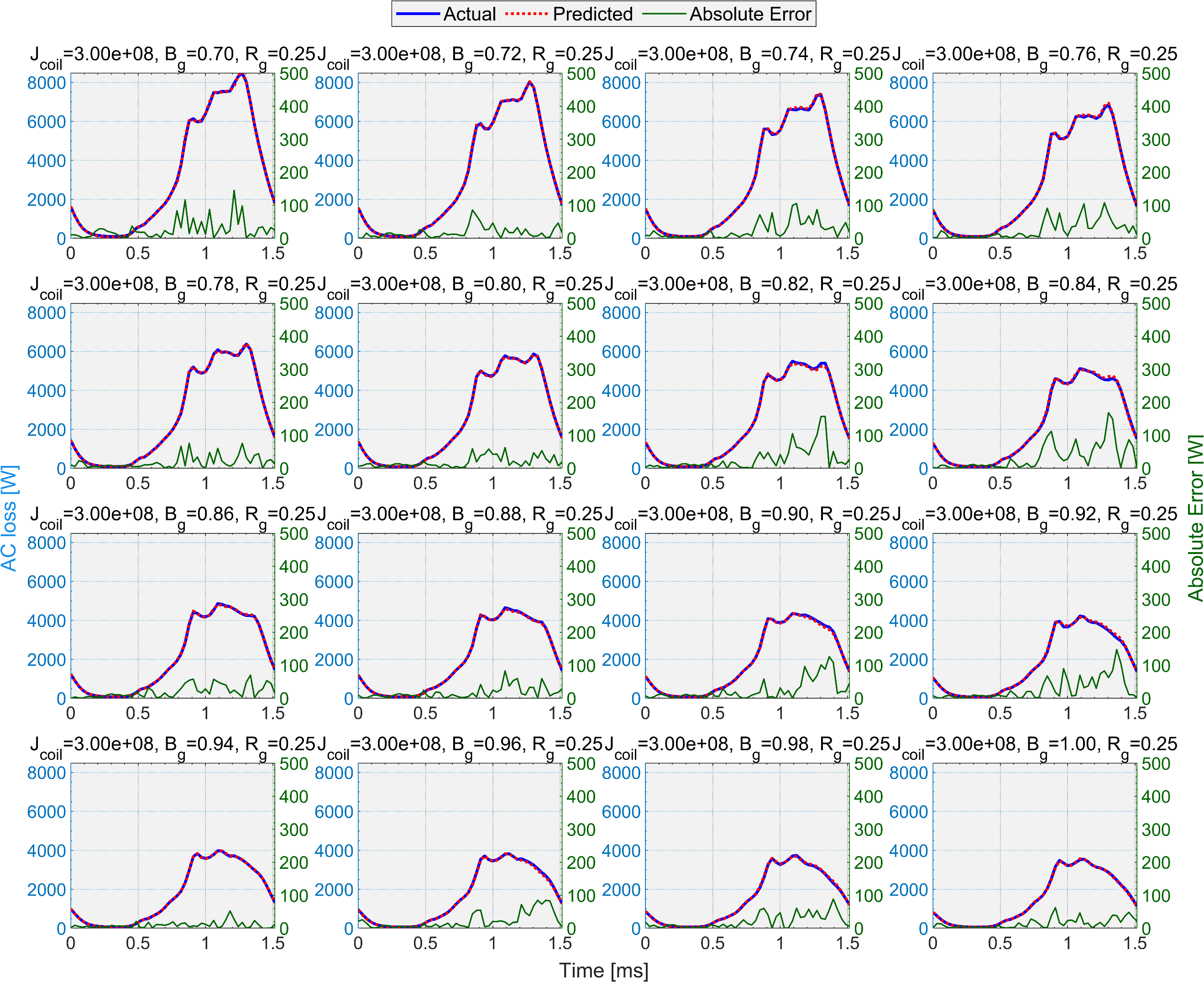


Figure S4. Comparison of the time-dependent AC loss of the motor with Different B_g_ and the predicted morphology of the CFNN model


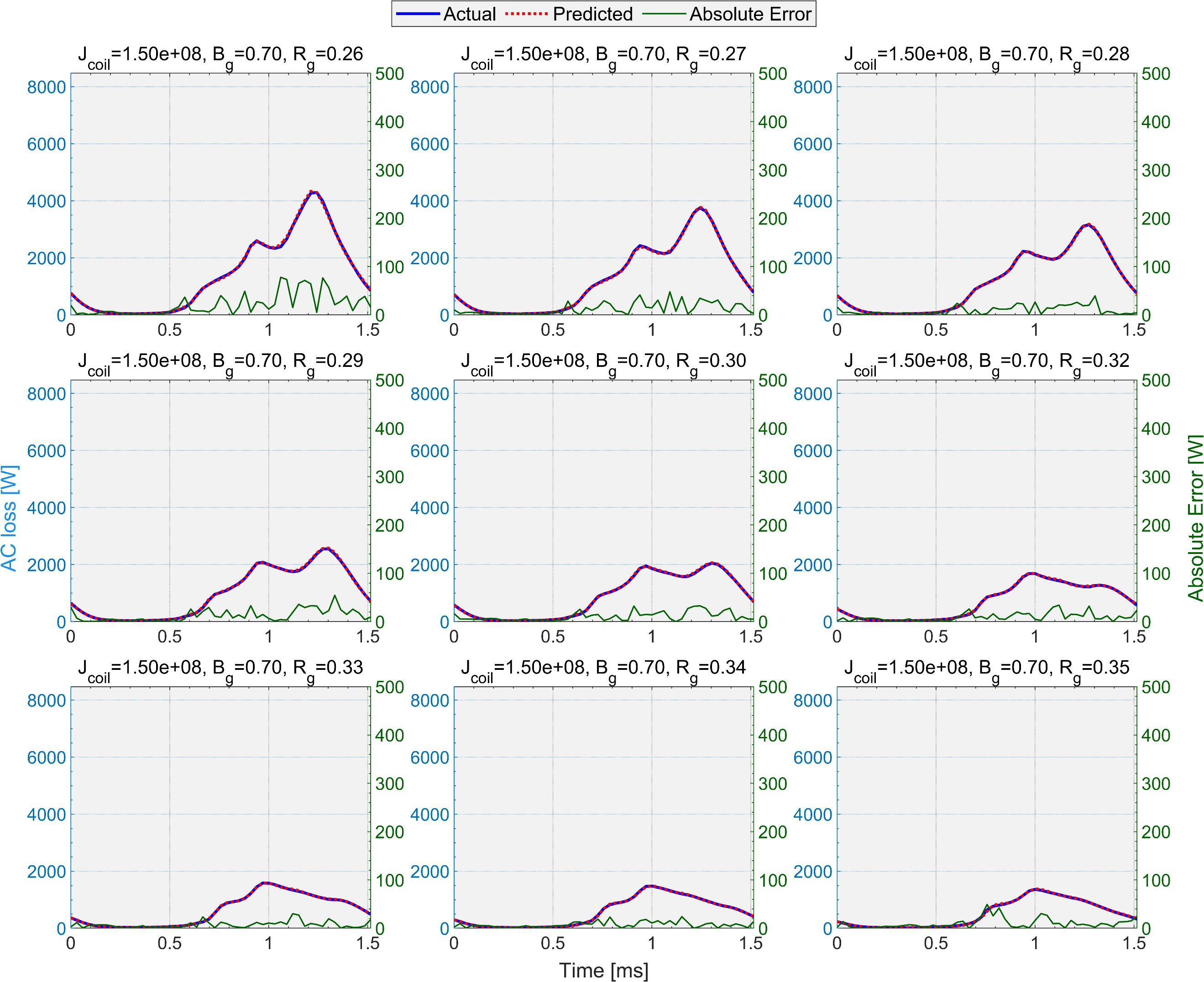


Figure S5. Comparison of the time-dependent AC loss of the motor with Different R_g_ and the predicted morphology of the CFNN model


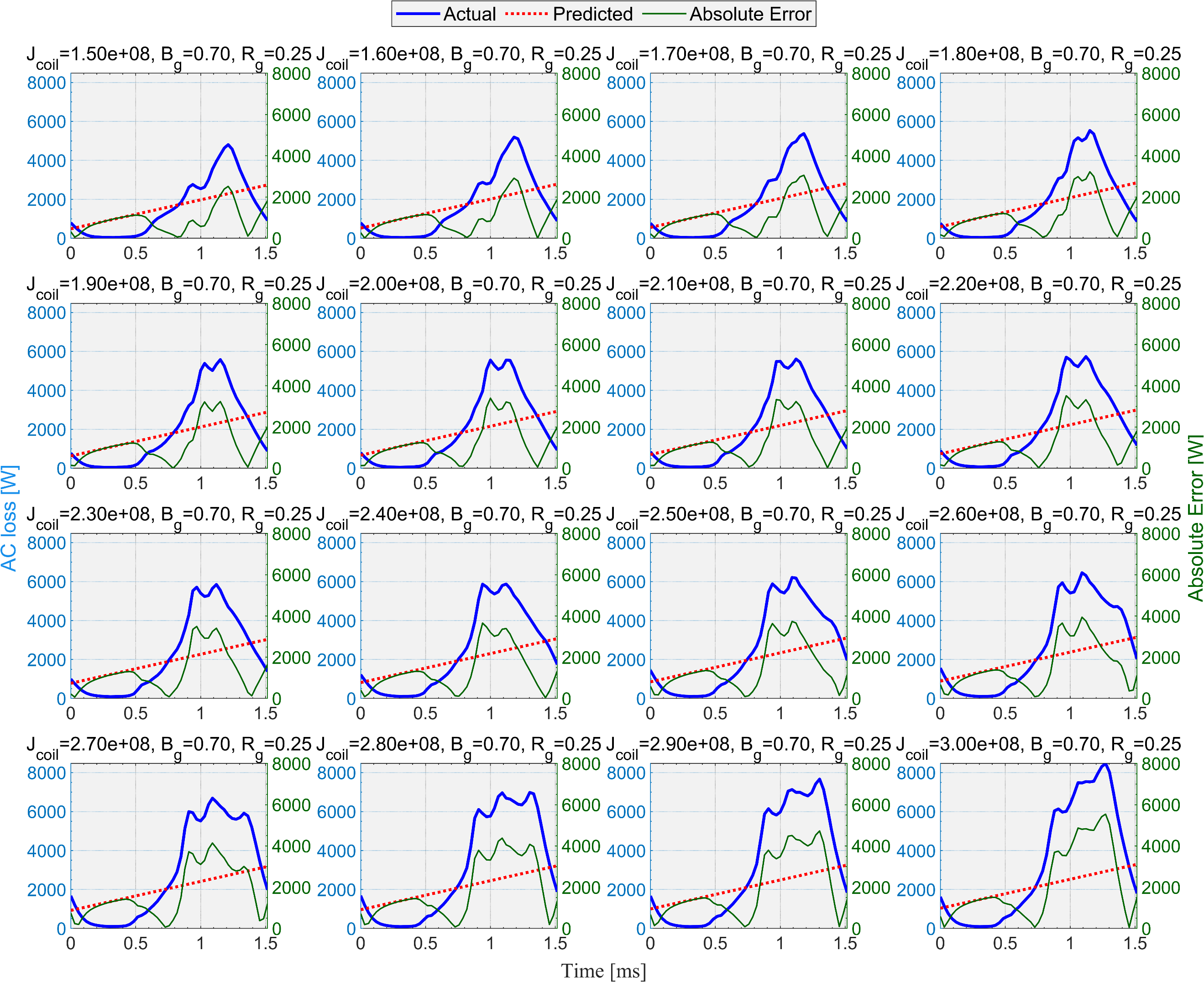


Figure S6. Comparison of the time-dependent AC loss of the motor with different J_coil_ and the predicted morphology of linear regression model


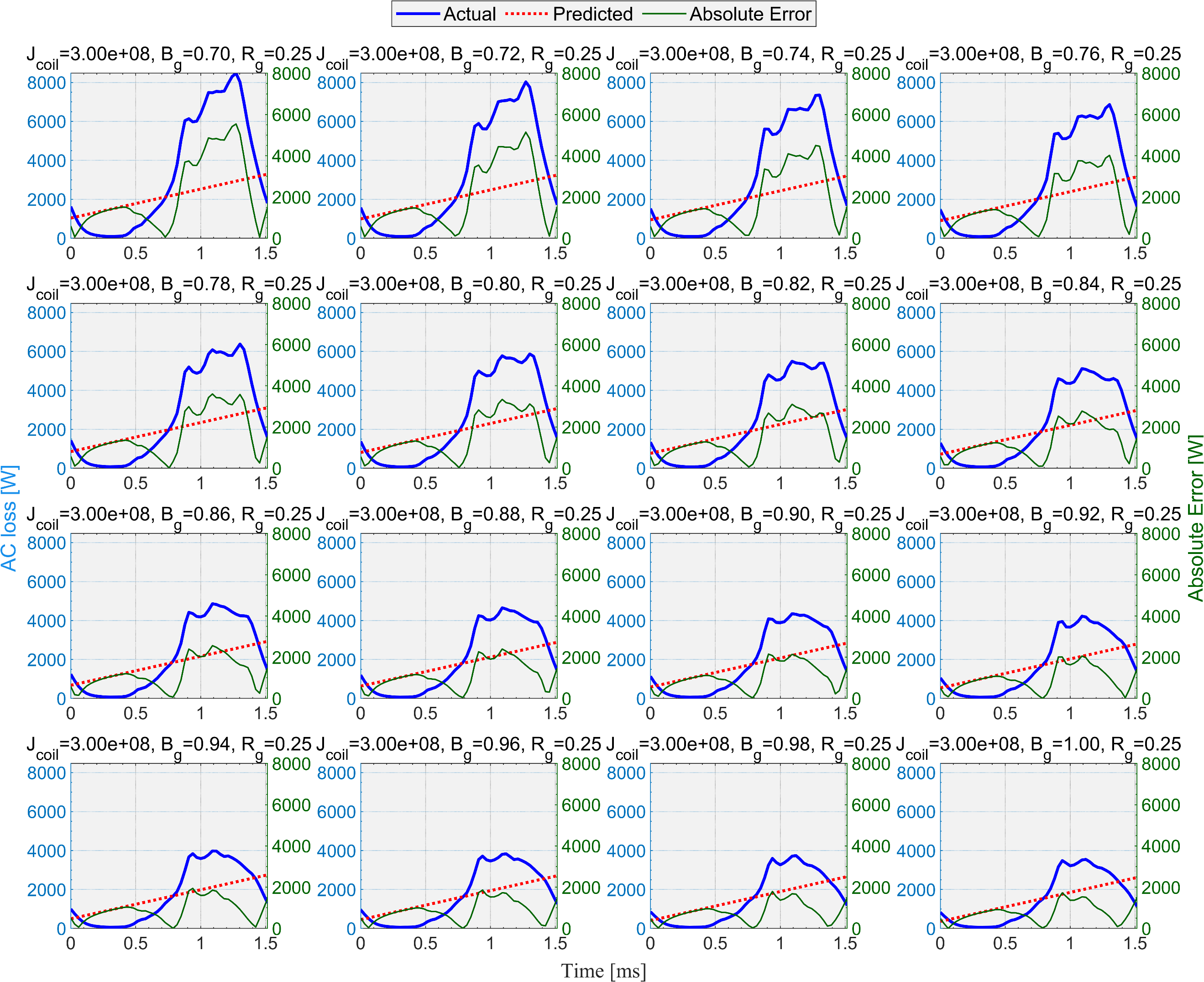


Figure S7. Comparison of the time-dependent AC loss of the motor with Different B_g_ and the predicted morphology of linear regression model


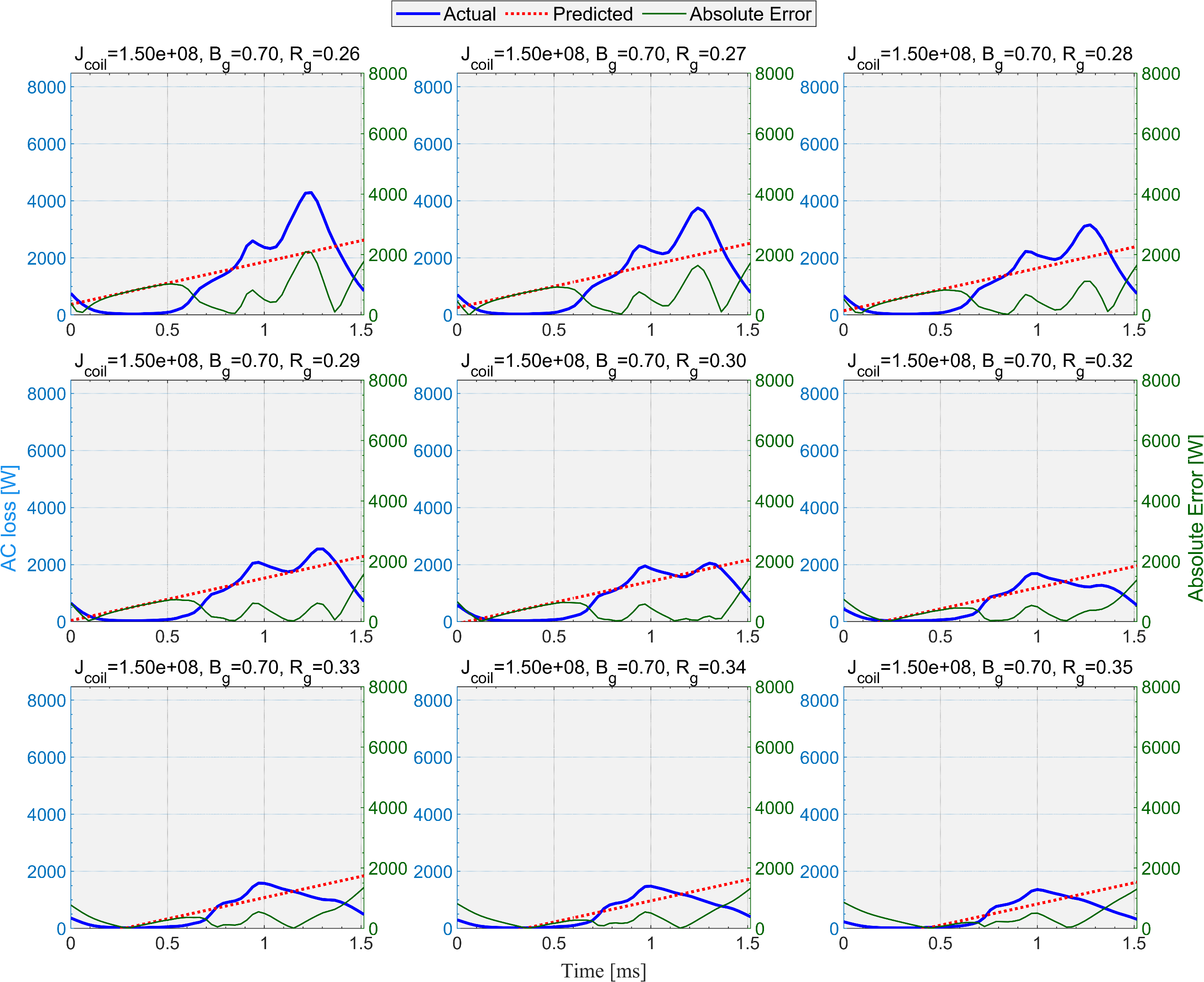


Figure S8. Comparison of the time-dependent AC loss of the motor with Different R_g_ and the predicted morphology of linear regression model

| **(a)**  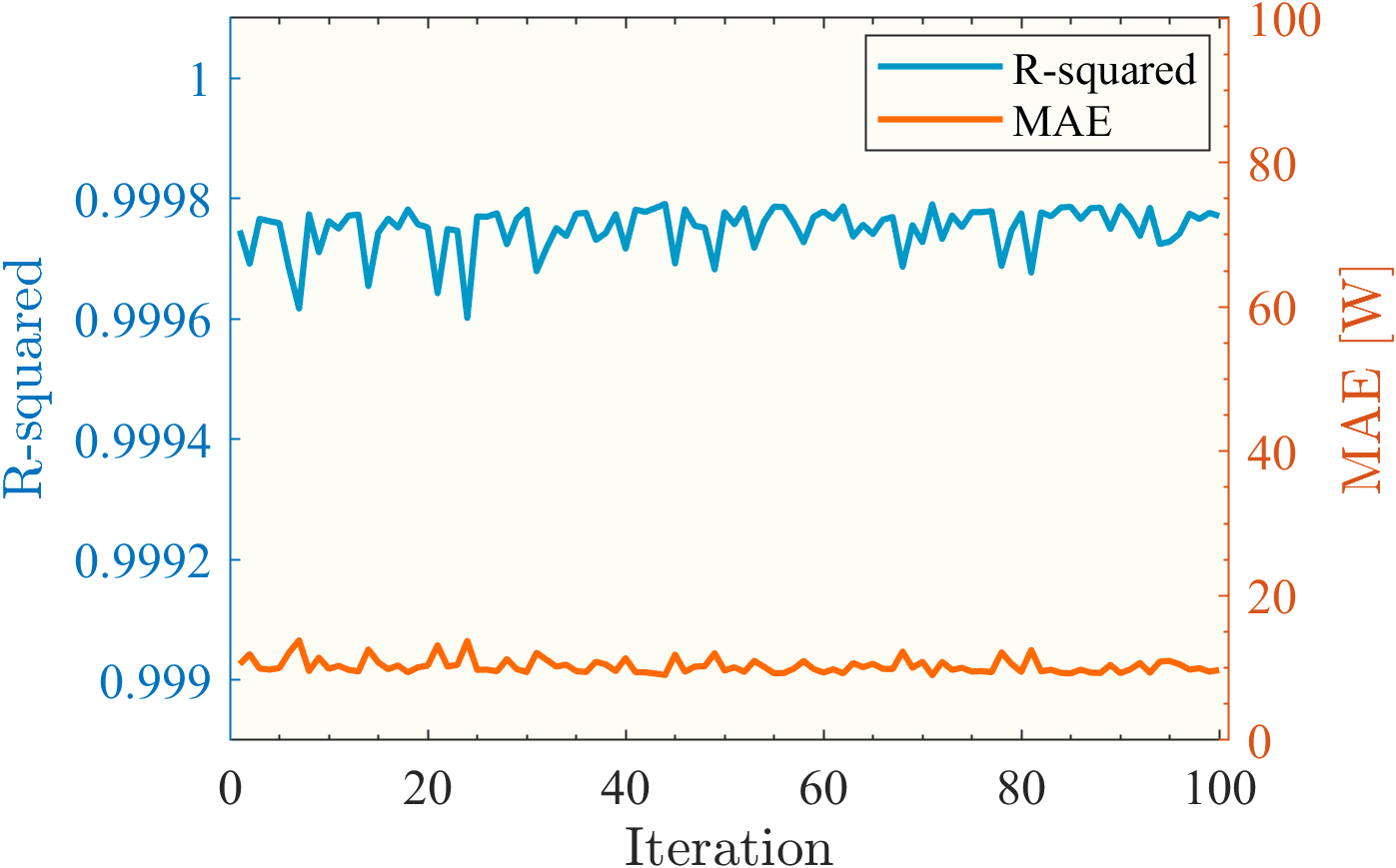 | **(b)**  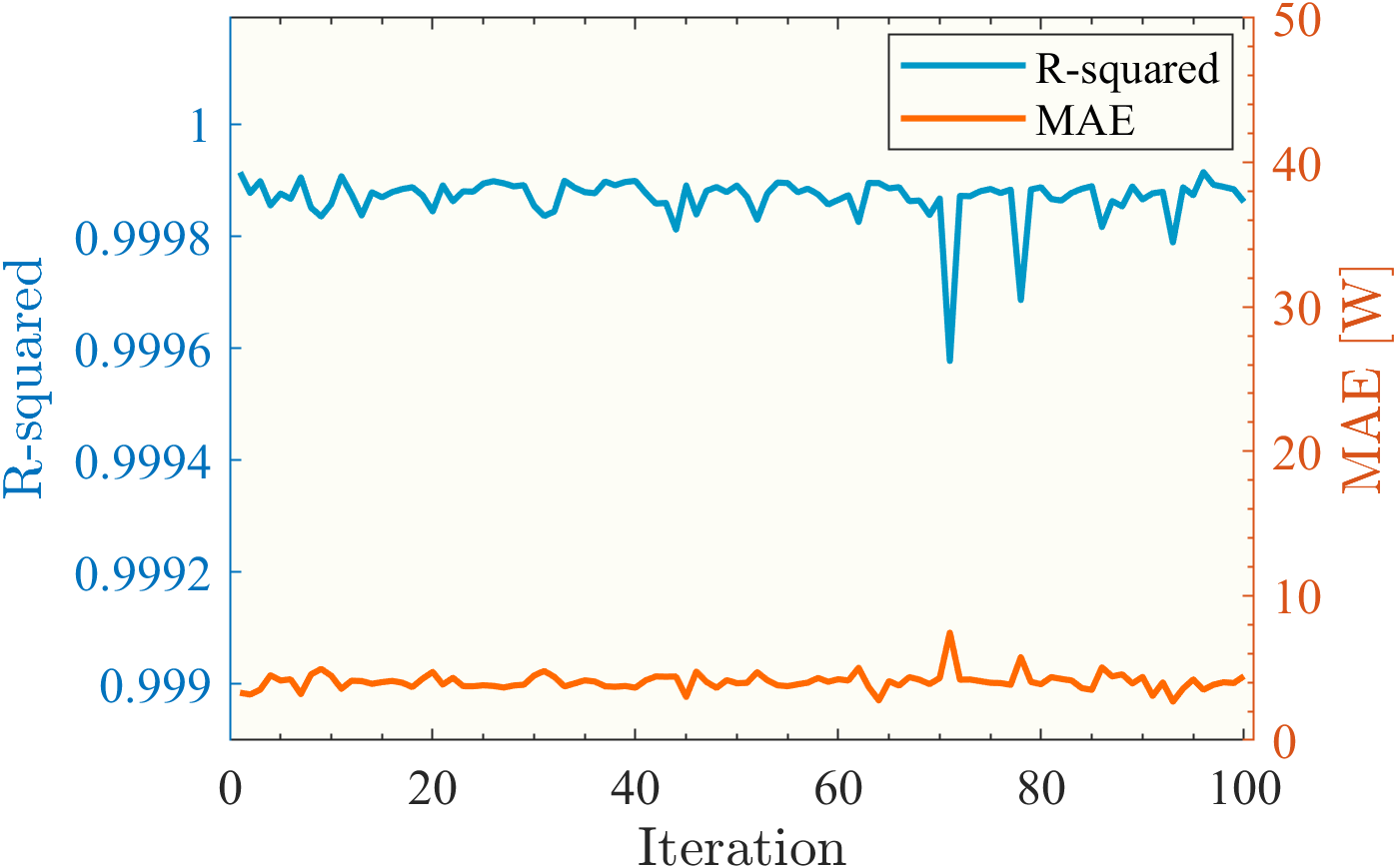 |
| --- | --- |

Figure S9. Stability of the results of the CFNN models. For dynamic (a) and static (b) losses

Table S4. The range of parameters for the new configurations of the motor used for extrapolation tests

| Parameter | B_g_ [T] | R_g_ [m] | J_coil_ [A/m^2^] | AR | Static Q_sc_ [W] | Dynamic Q_sc_ [W] |
| --- | --- | --- | --- | --- | --- | --- |
| Range | 0.6-0.7 & 1-1.1 | 0.25-0.35 | 1.3E8-1.5E8 & 3E8-3.2E8 | 0.3-0.4 | 304.94-5104.85 | 5.79-11638.36 |


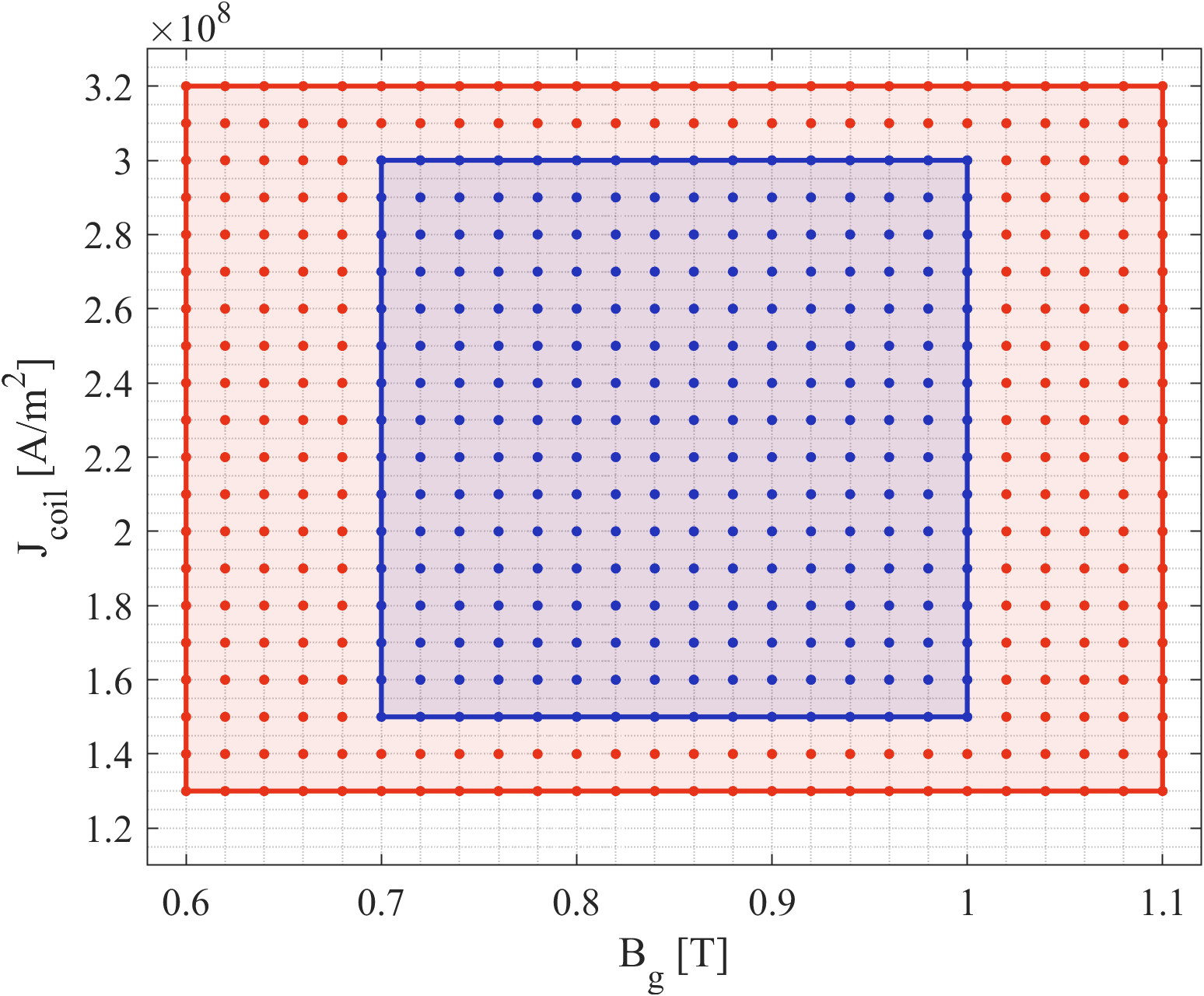


Figure S10. J_coil_ and B_g_ values for new parameters (red box) and for the training range of the CFNN model (blue box)

Figure S10 is also provided along with Table S4 in the supplementary information to visually show the new motor configurations in comparison to the trained data. In this figure, the blue dots represent the datapoints that existed in the initial dataset that was used for training the CFNN model, while red dots show the new motor configurations.

| **(a)**  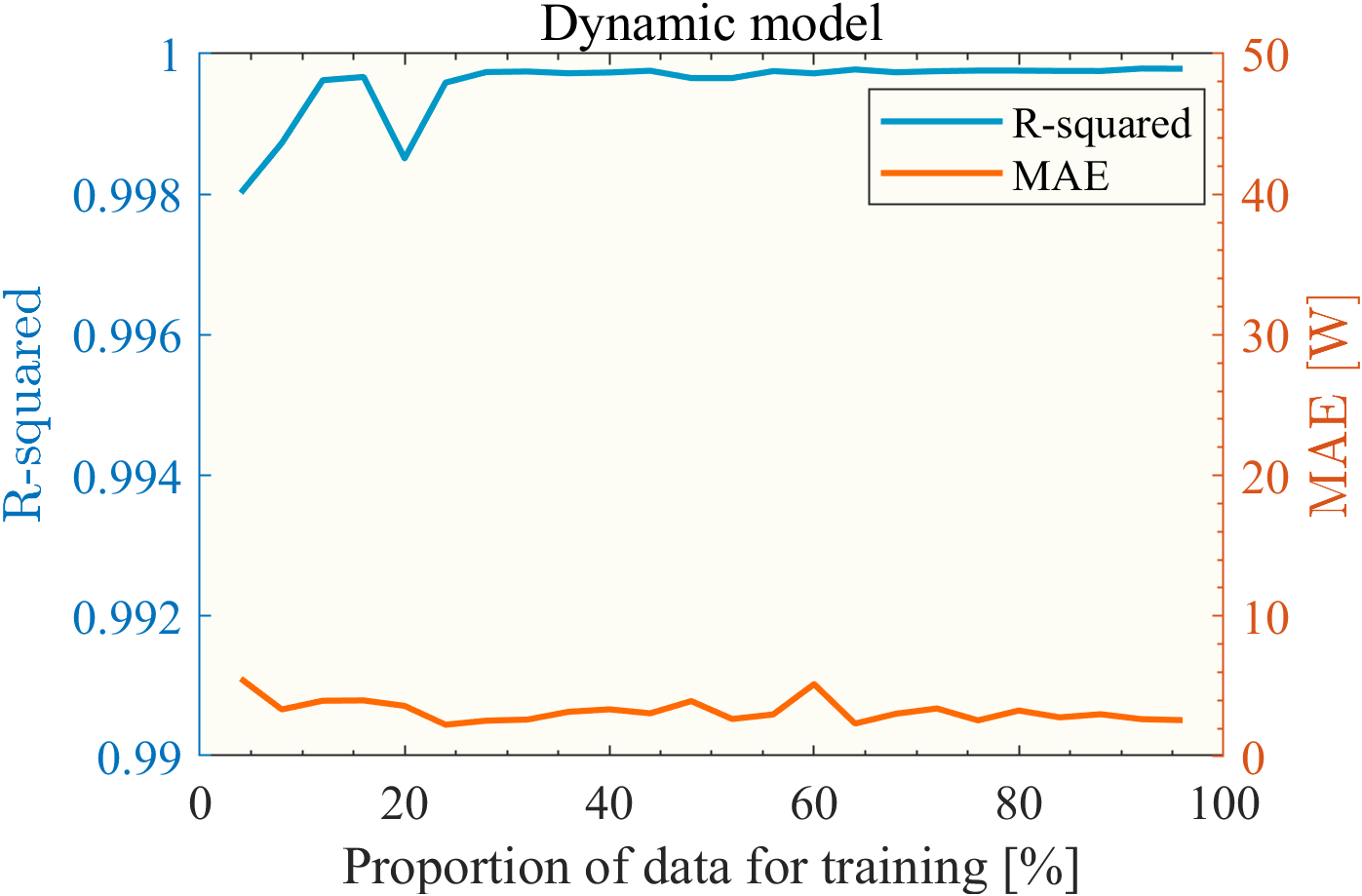 | **(b)**  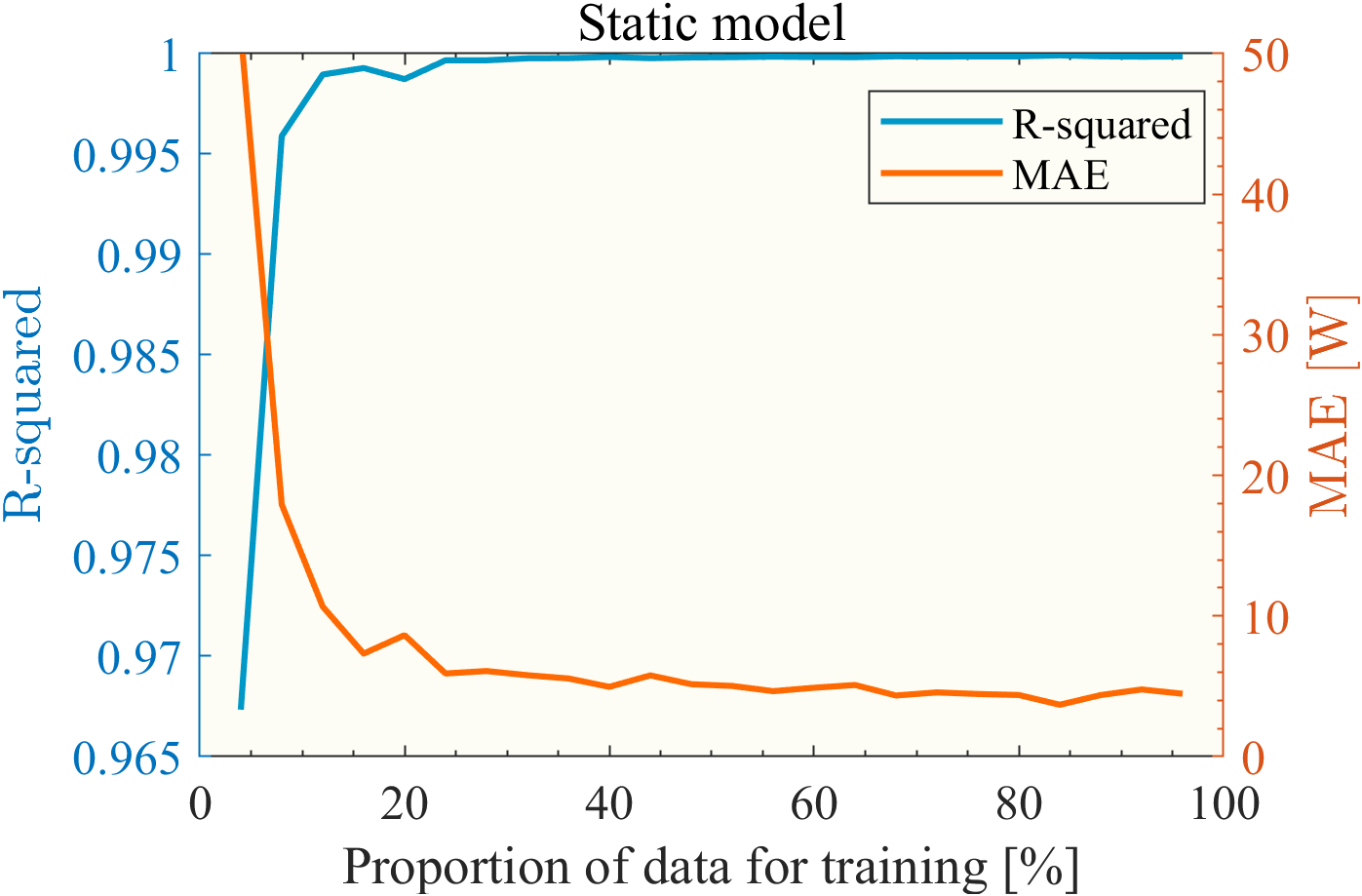 |
| --- | --- |

Figure S11. Performance of the CFNN model with different training set sizes

Table S5. Comparison of the simulation time and real-world time of the static and dynamic models in the Simulink environment

| Model | Virtual time of simulation (Simulink time) [s] | Real-world time of simulation (Actual time) [s] |
| --- | --- | --- |
| Static | 1 | 15.82 |
|  | 5 | 74.226 |
| Dynamic | 1 | 4.68 |
|  | 5 | 13.51 |

| **Algorithm S1: General algorithm for developing other surrogate model development** |
| --- |
| 1. **Import Data**   Load dataset → Randomly split into training and testing sets.   1. **Preprocess Data**   Normalize input and output variables → $X=\frac{x-x_{min}}{x_{max}-x_{min}}$  $Y=\frac{y-y_{min}}{y_{max}-y_{min}}$   1. **Initialize Model**   Define hyperparameters (e.g., depth, learning rate, neurons, kernel type).   1. **Train Model**   Define the loss function based on MSE → $MSE=\frac{1}{n}\sum_{i=1}^{n} \left( y_{i}-\hat{y_{i}} \right)^{2}$  Fit model on training data → Minimize MSE   1. **Test Model**   Predict outputs on test data using trained model.  Compute performance metrics (R^2^, RMSE, etc.).   1. **Optimize Model**   Change the hyperparameters to achieve optimal performance   1. **Output Results**   Display predicted vs actual values and model accuracy. |

| **Algorithm S2: CFNN surrogate model development algorithm** |
| --- |
| 1. **Import Data**   Load dataset → Randomly split into training and testing sets.   1. **Preprocess Data**   Normalize input and output variables → $X=\frac{x-x_{min}}{x_{max}-x_{min}}$  $Y=\frac{y-y_{min}}{y_{max}-y_{min}}$   1. **Initialize Network**   Define CFNN with:  Input layer (features) → $y_{i}=x_{i}$  Hidden layer(s) with nonlinear activation → $y_{k}=f_{j}^{H}\left( \sum_{i=1}^{n} \omega_{jh}^{H}x_{i} \right)$  Cascade connections → $f^{0} \left( \omega^{b}+\sum_{j=1}^{n} \omega_{i}^{0}x^{i}f_{j}^{H}\left( \omega_{i}^{0}+\sum_{i=1}^{n} \omega_{jh}^{H}x_{i} \right) \right)$ so each layer connects to all previous layers  Output layer (linear activation) → $y_{p}=\sum_{i=1}^{n} f^{i}\omega_{i}^{0}x^{i}+f^{0} \left( \omega^{b}+\sum_{j=1}^{n} \omega_{i}^{0}x^{i}f_{j}^{H}\left( \omega_{i}^{0}+\sum_{i=1}^{n} \omega_{jh}^{H}x_{i} \right) \right)$   1. **Train Model**   Define the loss function based on MSE → $MSE=\frac{1}{n}\sum_{i=1}^{n} \left( y_{i}-\hat{y_{i}} \right)^{2}$  **For** i = 1 to max_epochs do  **\|** Use Levenberg–Marquardt optimization algorithm to minimize MSE.  **\|** Calculate the gradient of changes in MSE → $Grad=MSE_{i}-MSE_{i-1}$  **\| If** $Grad<\mu$  **\|** **\|** Break the loop  **\| Else if** $Grad\_i>0$ for 10 Epochs  **\| \|** Break the loop  **\| End**  **End**   1. **Test Model**   Predict outputs on test data using trained model.  Compute performance metrics (R^2^, RMSE, etc.).   1. **Optimize Model**   Change the depth and length of the network to achieve optimal performance   1. **Output Results**   Display predicted vs actual values and model accuracy. |
